# Pharmacological interventions to prevent cardiotoxicity in patients undergoing anthracycline-based chemotherapy: a network meta-analysis

**DOI:** 10.3389/fcvm.2025.1612060

**Published:** 2025-09-03

**Authors:** Xiaoyu Liu, Ran Ding, Aihua Zhang

**Affiliations:** School of Nursing, Shandong First Medical University & Shandong Academy of Medical Sciences, Taian, China

**Keywords:** anthracyclines, pharmacological intervention, cardiotoxicity, chemotherapy, tumors

## Abstract

**Background:**

This study evaluates the efficacy of various pharmacological therapies in mitigating the cardiotoxicity associated with anthracycline chemotherapy and furnishes contemporary, evidence-based guidelines and recommendations for clinical practice.

**Methods:**

We searched the EMBASE, Cochrane Library, PubMed, Web of Science, and Scopus databases from the beginning of each database to April 2024 and were limited to English-language documents. The primary objective of this study is to assess the efficacy of cardioprotective drugs in preventing the reduction of left ventricular ejection fraction (LVEF) and the incidence of cardiac events. The secondary objective is to evaluate the impact of these drugs on reducing left ventricular end-systolic diameter (LVESD) and left ventricular end-diastolic diameter (LVEDD), as well as on maintaining the ratio of peak mitral annular diastolic velocity to atrial contraction velocity (E/A ratio).

**Results:**

54,852 studies were retrieved from five databases, and 28 randomized controlled trials involving 2,858 patients were finally included. Network Meta-analysis results showed that, compared to the control group, Spironolactone demonstrated the most significant improvement in (LVEF [MD = 12.10, 95% CI (7.50, 16.70)] and LVESD [MD = −5.00, 95% CI (−7.68, −2.32)]. For reducing cardiac events, Dexrazoxane [OR = 0.28, 95% CI (0.16, 0.50)] and Vitamin E combined with Levocarnitine [OR = 0.27, 95% CI (0.08, 0.90)] were the most effective interventions. In terms of diastolic function (E/A ratio), Nebivolol outperformed other β-blockers [MD = 0.23, 95% CI (0.09, 0.37)]. However, no intervention demonstrated a statistically significant effect on LVEDD.

**Conclusion:**

According to the research findings, Spironolactone and Dexrazoxane significantly prevent the decline in LVEF and the occurrence of cardiac events compared to placebo or conventional chemotherapy, with statistical significance. This discovery provides valuable reference for the clinical prevention of anthracycline chemotherapy-induced cardiotoxicity, contributing to the optimization of treatment regimens, reduction of cardiac toxicity risks in patients, and improvement of prognosis.

**Systematic Review Registration:**

PROSPERO (CRD42024567684).

## 1 Introduction

Cancer represents one of the most significant burdens of public health as a leading cause of mortality worldwide. Chemotherapy plays a prominent role in cancer treatment, particularly with anthracyclines, which are extensively utilized for both solid tumors and hematological malignancies due to their potent anti-tumor capability, including in breast cancer, lung cancer, lymphoma, and leukemia (1). However, the cardiotoxicity associated with anthracyclines is still considered by practitioners to be a substantial challenge in clinical practice. Congestive heart failure has become a primary cause of morbidity and mortality in cancer survivors, negatively impacting treatment outcomes and potentially jeopardizing their long-term quality of life (2, 3).

Anthracyclines and their derivative compounds (such as doxorubicin, idarubicin, epirubicin, and mitoxantrone) constitute a class of chemotherapeutic drugs known for their significant cardiotoxicity (4). This cardiotoxicity is primarily characterized by dose-related, delayed, and irreversible heart failure (5), which can result in various complications, including coronary artery disease, arrhythmias, pericardial disease, valvular disease, myocardial fibrosis, and left ventricular dysfunction (6, 7). Anthracyclines increase the risk of clinical cardiotoxicity by 5.43 times, subclinical cardiotoxicity by 6.25 times, any cardiotoxicity by 2.27 times, and cardiac mortality by 4.94 times compared to non-anthracycline regimens (8). The cardiotoxic effects of anthracyclines are progressive and irreversible, potentially causing myocardial damage even with initial administration (9). As the cumulative dose increases, the risk of cardiotoxicity escalates significantly (10, 11). Furthermore, cardiotoxicity is not confined to acute-phase reactions; it also encompasses chronic and delayed cardiac adverse effects, which may manifest years after the cessation of chemotherapy, thereby heightening the long-term health risks for patients (12). Although the implementation of liposome technology can mitigate cardiotoxicity to some extent, certain risks remain (13). Additionally, anthracyclines are associated with severe dose-dependent acute toxicity, which restricts their long-term repeated use.

In recent years, numerous studies have explored various pharmacological intervention strategies to reduce cardiotoxicity associated with anthracycline chemotherapy (14). Prophylactic dexrazoxane is the sole pharmacological agent sanctioned for the clinical prevention and management of anthracycline-induced cardiotoxicity (15). Studies have shown that combining dexrazoxane and anthracyclines can significantly mitigate cardiotoxicity (16). Additionally, beta-blockers, angiotensin-converting enzyme (ACE) inhibitors, and angiotensin II receptor blockers (ARBs) are also considered to possess potential cardioprotective effects (17). This study seeks to update previous research by integrating the most recent data to provide evidence-based guidance and recommendations for preventing cardiotoxicity from anthracycline chemotherapy.

## 2 Methods

This review is presented in compliance with the Preferred Reporting Items for Systematic Reviews and Meta-Analyses Extended Statement, and our review is registered with PROSPERO (CRD42024567684) (18).

### 2.1 Data sources and searches

We performed an extensive systematic literature search, covering EMBASE, Cochrane Library, PubMed, Web of Science, and Scopus databases, to collect all randomized controlled trials on pharmacological intervention to improve the cardiotoxicity of anthracycline chemotherapy drugs. Documents restricted to the English language were considered, and the search period was from the inception of each database to April 2024. The initial screening employed a search approach that integrated subject headings with free words tailored to the particularities of various databases, using keywords such as “anthracyclines” and “cardiotoxicity.” The search results were imported into Endnote ×9 for document organization and classification to enable deduplication and cross-validation. Details on specific search strategies are provided in the Supplementary S1.

### 2.2 Trial selection criteria and trial identification

Following the elimination of duplicates, titles and abstracts were separately evaluated by two researchers (Liu XY, DR) according to established inclusion and exclusion criteria, with any discrepancies rigorously addressed through discussion. The following inclusion criteria were adopted: (a) patients diagnosed with malignant tumors and planned to receive anthracycline-based chemotherapy; (b) randomized clinical trials (RCTs) assessing the prophylactic impact of pharmacological intervention on anthracycline-induced cardiotoxicity; (c) No previous heart disease and no use of heart-related drugs; The following exclusion criteria are used: (a) studies in languages other than English; (b) patients with any other condition that causes taking beta-blockers, ACE inhibitors, ARBs or statins Patients with concomitant diseases such as drugs; (c) Patients with active heart disease, including myocardial infarction, active angina, symptomatic valvular heart disease, or uncontrolled congestive heart failure within 12 months; (d) Patients with diabetes.

### 2.3 Outcomes and data extraction

Primary Outcome Measures: The primary outcome of this study is to evaluate the impact of cardioprotective drugs on left ventricular ejection fraction (LVEF), comparing the LVEF between the intervention group and the control group, and assessing whether cardioprotective drugs can effectively prevent the decline of LVEF. Additionally, the incidence of cardiac events in both groups of patients will be recorded and compared. A cardiac event is defined as the occurrence of any of the following: a reduction in LVEF by more than 10% or an absolute decrease in LVEF to below 40%; the emergence of clinical signs of heart failure.

Secondary Outcome Measures: The changes in left ventricular end-systolic diameter (LVESD) and left ventricular end-diastolic diameter (LVEDD) will be compared to assess whether cardioprotective drugs help reduce these parameters and alleviate cardiac dilation. The ratio of peak diastolic velocity of the mitral annulus to the velocity of atrial contraction (E/A ratio) is an important indicator for evaluating left ventricular diastolic function. We will compare the E/A ratio of patients to assess the effectiveness of cardioprotective drugs in enhancing early diastolic filling capacity of the heart.

Two researchers created an Excel table for literature information extraction to independently perform literature screening and data extraction based on the inclusion and exclusion criteria, followed by cross-verification. If there were any differences, the decision was made through analysis and discussion by two people or a joint conversation with a third researcher. The following parameters were extracted: (1) Study characteristics: first author's name, publication year, clinicaltrials. gov trial number, intervention, and comparison, follow-up duration; (2) Participants' baseline characteristics: total number of participants, mean age, percentage of women; (3) Intervention details: intervention name, dosing regimen. Data on the mean and standard deviation (SD) were extracted for continuous outcomes, and the number of participants in which an event occurred was extracted for binary outcomes. Only the primary study was considered if the same population participated in more than one published study.

### 2.4 Quality and risk of bias assessment

The Cochrane Collaboration RCT risk of bias assessment tool ROB2.0 (https://www.riskofbias.info/welcome/rob-2-0-tool) was used to assess the risk of bias (19). Two researchers independently assessed the risk of bias in the included studies and cross-checked the results. It consists of the following six parts: (1) Bias generated during the randomization process; (2) Bias deviating from the established intervention; (3) Bias due to missing outcome data; (4) Bias in outcome measurement; (5) Result selectivity reporting bias. According to the manual standards, the risk of bias in each module can be divided into three levels: “low risk,” “some concerns,” or “high risk.” If there are discrepancies in the results, the decision will be made through discussion with a third researcher.

### 2.5 Data synthesis and analysis

The Mvmeta package and Network package in Stata18 and related commands are used to calculate and process the data. The effect size of the dichotomous outcomes uses the odds ratio (OR), and mean difference (MD) was chosen as a standardized pooled effect size in pairwise comparisons. Then, draw an evidence network diagram in which each node represents an intervention, and the size of the node reflects the sample size of the corresponding intervention; the connection between nodes indicates a direct comparison between the two interventions, and the thickness of the connection demonstrates the amount of direct comparison evidence. The inconsistency test is performed through the node-splitting method. If the test results show that the difference is not statistically significant (*P* > 0.05), the consistency model is used for analysis, and the results are sorted; otherwise, the non-consistency model is used for analysis. Owing to the absence of closed loops, global and local inconsistency tests were not performed. To evaluate the effectiveness of each intervention, the surface under the cumulative ranking curves (SUCRA) was used to rank, and forest plots and league tables were drawn for each intervention to compare with placebo. In addition, publication bias and heterogeneity were analyzed using funnel plots.

## 3 Results

### 3.1 Search results

**Figure 1:**
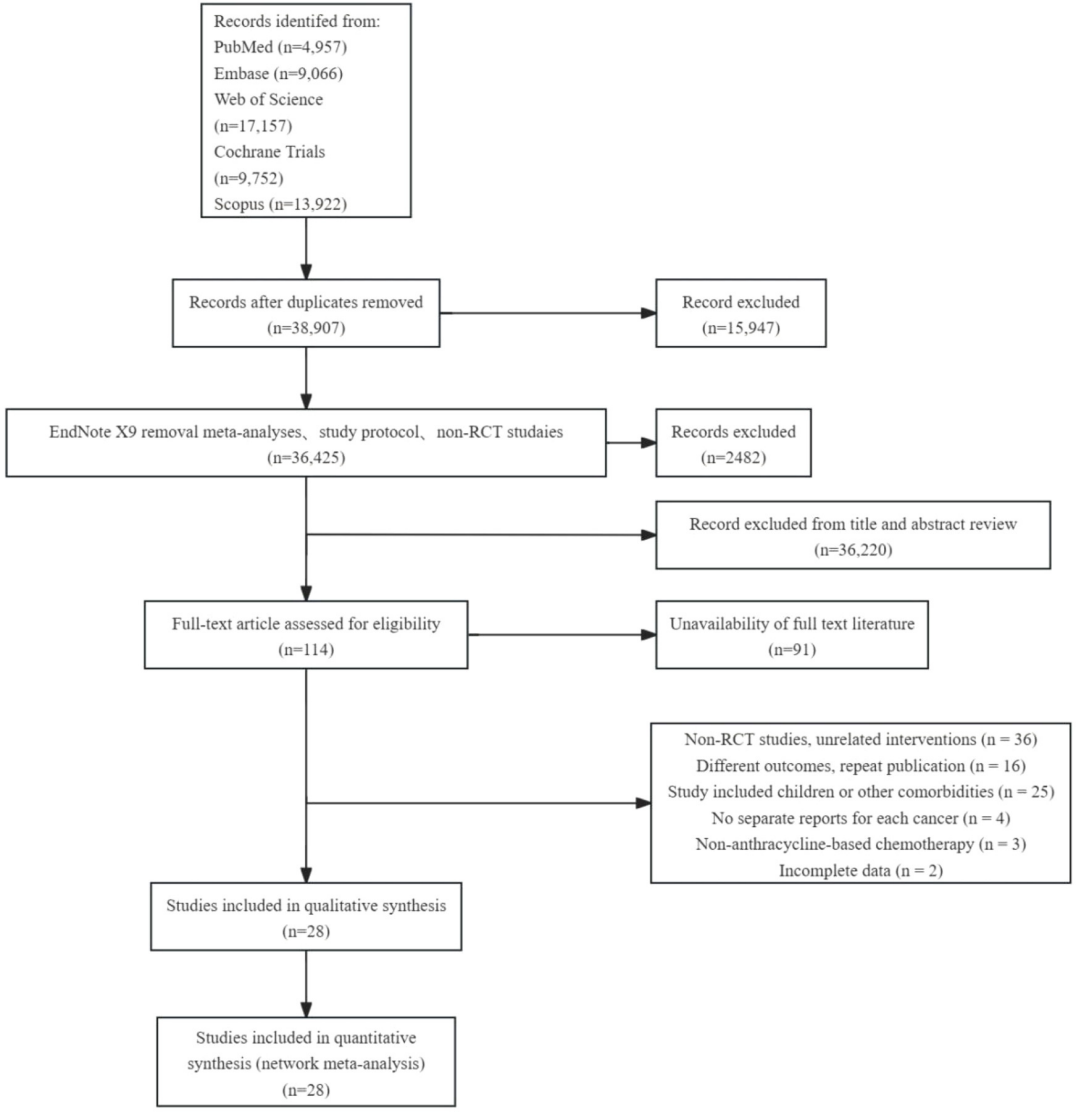
Flow diagram of the study selection process for this network meta-analysis.

54,852 studies were retrieved from five databases: EMBASE, Cochrane Library, PubMed, Web of Science, and Scopus. After deleting duplicate documents and excluding non-randomized controlled trials (RCT), 36,425 articles remained. By screening titles and abstracts, we retained 206 papers relevant to this study, of which the full text was available for 117 papers. After applying the inclusion and exclusion criteria for full-text re-screening and excluding unqualified documents, 28 papers were finally included in our analysis. The literature screening process and results are shown in Figure 1.

### 3.2 Characteristics of the included trials

Twenty-eight studies had inclusion criteria and were published between 1987 and 2024 with 2,858 patients, including 1,435 in the intervention group and 1,423 in the control group. A total of 22 drugs were included in the studies, including three articles on Dexrazoxane, two articles on Enalapril, four articles on Carvedilol, two articles on Nebivolol, and ICRF-187, Metoprolol, N-Acetylcysteine, Lisinopril and Bisoprolol, Astragalus polysaccharide, Ivabradine, Combined treatment with carvedilol and candesartan, Metformin, Vitamin E and levocarnitine, Prenylamine, Telmisartan, Salidroside, Spironolactone, Platycodon grandiflorum, Hong Huang Decoction, Alpha-lipoic acid, Panax ginseng, and Atorvastatin. The basic information of the included articles is shown in Supplementary S4-S5.

### 3.3 Risk of bias in included studies

Overall, 13 of the 28 randomized controlled studies were rated as “high risk,” and five were rated as 'some concerns.' Regarding random sequence generation methods, 11 studies only mentioned randomization but did not specify how randomization is achieved; therefore, they are rated as 'some concerns'; the rest are low risk. Regarding deviation from the intended intervention, 13 studies did not use a placebo or sham intervention in the control group and were therefore rated as “high risk.” (Supplementary S6).

### 3.4 Primary outcomes

#### 3.4.1 LVEF

**Figure 2:**
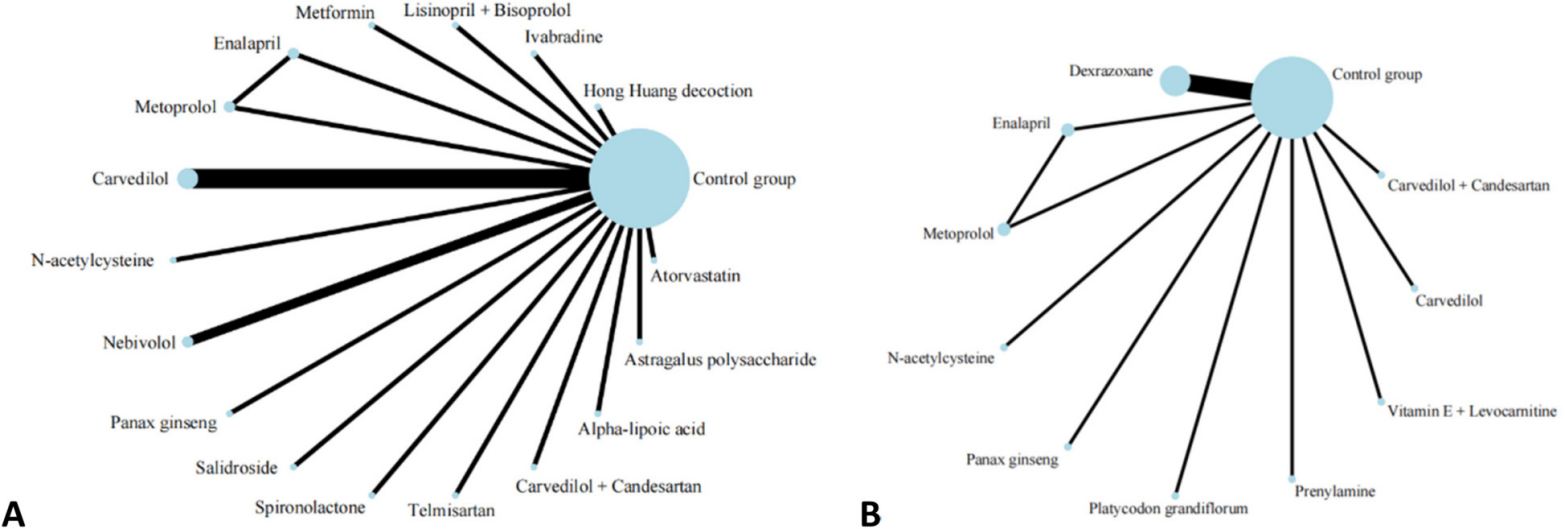
Evidence network of A and B in the meta-analysis. **(A)** left ventricular ejection fraction; **(B)** cardiac events.

A total of 20 studies investigated the impact of pharmacological interventions on preventing or alleviating the decline in left ventricular ejection fraction caused by anthracycline chemotherapy drugs, encompassing 18 different types of interventions. The evidence network diagram (Figure 2) indicates that carvedilol has the largest sample size and the most extensive literature analyzed compared to the control group. Among the assessed interventions (Supplementary S7), Spironolactone demonstrated the strongest improvement in LVEF [MD = 12.10, 95% CI (7.50, 16.70)]. This was followed by the combination therapy of Lisinopril and Bisoprolol [MD = 5.38, 95% CI (0.32, 10.44)] and Astragalus polysaccharide [MD = 4.24, 95% CI (0.10, 8.38)], both of which exhibited statistically significant positive effects. Conversely, another traditional Chinese medicine, Panax ginseng [MD = −6.90, 95% CI (−10.46, −3.34)], was the only intervention to show a significant negative effect, suggesting that ginseng may have a potential adverse impact on the prevention of LVEF decline. According to the SUCRA values sorted by effect size and probability, it can be seen that Spironolactone (99.8%) >Lisinopril + Bisoprolol (84.6%)  > Astragalus polysaccharide (79.4%) > Metformin (76.2%) > Nebivolol(73.1%) > Atorvastatin (70.2%) > Carvedilol (53.0%) > Telmisartan (51.7%) > Carvedilol + Candesartan (49.6%) > Salidrosid (41.8%) > Control group (39.8%) > N-acetylcysteine (37.0%) > Hong Huang decoction (36.7%) > Alpha-lipoic acid (36.4%) > Ivabradine(33.1%) > Enalapril(19.8%) > Metoprolo l(16.0%) > Panax ginseng (1.8%).

#### 3.4.2 Cardiac events

**Figure 3:**
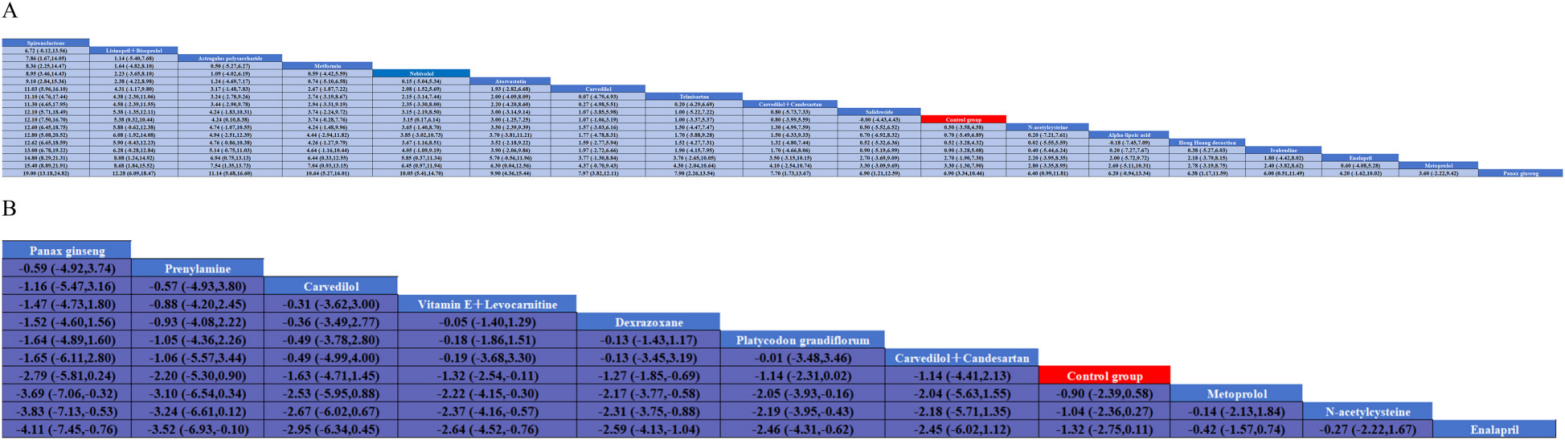
Pairwise meta-analysis results for A and B. **(A)** left ventricular ejection fraction; **(B)** cardiac eventscardiac events.

Thirteen investigations documented the impact of pharmacological intervention on decreasing the occurrence of cardiac events induced by anthracycline chemotherapeutic agents, covering 11 types of interventions (Figure 2) The pairwise comparison results of interventions showed that Dexrazoxane was superior to Enalapril, N-acetylcysteine, and Metoprolol in reducing the incidence of cardiac events (Figure 3). Panax ginseng and Vitamin E + Levocarnitine demonstrated better efficacy than Enalapril; while Platycodon grandiflorum, Panax ginseng, and Vitamin E + Levocarnitine were more effective than both Metoprolol and N-acetylcysteine. The forest plot results compared with the control group indicated that Dexrazoxane [OR = 0.28, 95% CI (0.16,0.50)] and Vitamin E + Levocarnitine [OR = 0.27, 95% CI (0.08,0.90)] were effective interventions for reducing the incidence of cardiac events in patients. SUCRA sorting can be known that Panax ginseng (84.3%) > Prenylamine (75.0%) > Vitamin E + Levocarnitine (67.5%) > Dexrazoxane (66.5%) > Carvedilol(65.2%) > Platycodon grandiflorum (62.9%) > Carvedilol + Candesartan (51.7%)> Control group (35.2%) > Metoprolol (18.0%) > N-acetylcysteine (14.4%) > Enalapril(9.2%) (Supplementary S7, S8).

**Figure 4:**
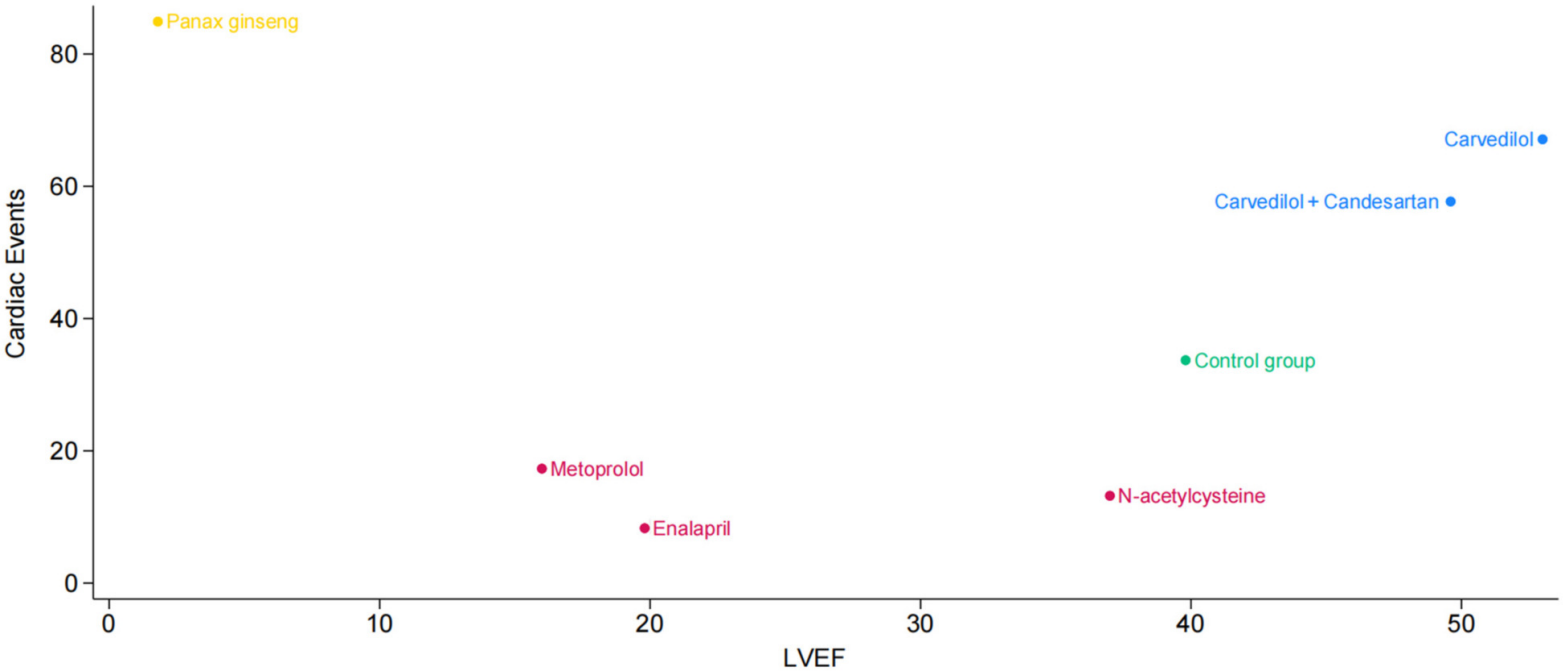
Cluster ranking diagram of left ventricular ejection fraction and cardiac events.

Additionally, we plotted a cluster ranking diagram of LVEF and cardiac events to identify pharmacological interventions that can effectively maintain LVEF while reducing the incidence of cardiac events. Figure 4 depicts the horizontal axis as LVEF and the vertical axis as cardiac events. Each point corresponds to a treatment method, with its color and position reflecting the impact on cardiac events and LVEF. The points for Carvedilol and Carvedilol + Candesartan are located in the upper right quadrant, indicating that these two drug interventions not only demonstrate outstanding performance in improving LVEF but also effectively reduce the incidence of cardiac events, showcasing favorable comprehensive efficacy. N-acetylcysteine is positioned in the middle-right section of the graph, suggesting it may have some positive impact on LVEF but exhibits relatively weaker effects in reducing cardiac event risks. Panax ginseng is situated in the upper left corner, implying it may possess certain potential in lowering cardiac event risks, though its effect on improving LVEF appears to be somewhat limited.

### 3.5 Secondary outcomes

#### 3.5.1 LVESD

**Figure 5:**
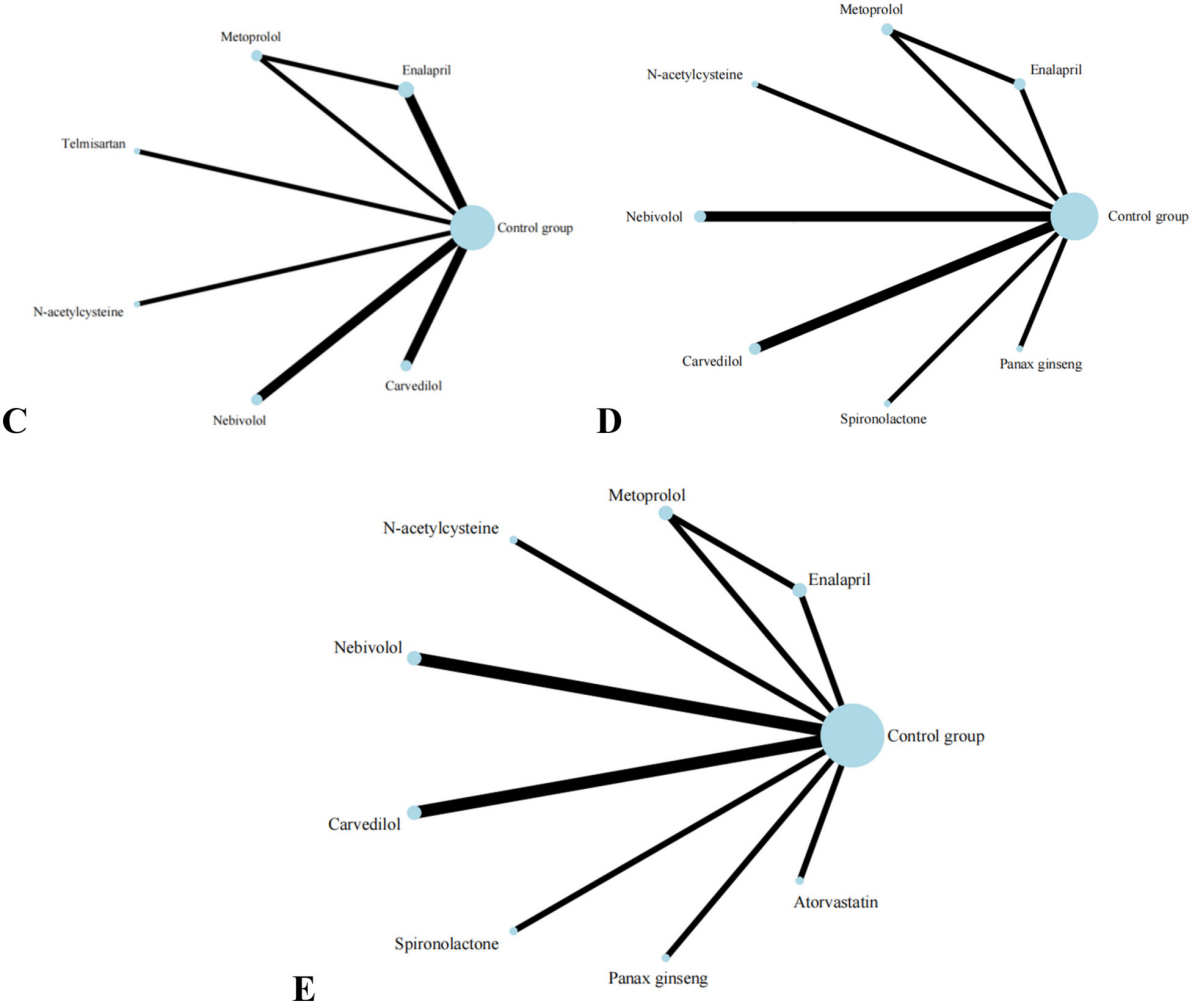
Evidence network of C, D, and E in the meta-analysis. **(C)** the ratio of mitral annular diastolic peak velocity to atrial systolic velocity; **(D)** left ventricular end-diastolic diameter; **(E)** left ventricular end-diastolic diameter.

Nine studies reported on LVESD, and the results included nine interventions (Figure 5). The contingency table analysis based on pairwise comparisons between interventions demonstrated that Spironolactone exhibited superior efficacy compared to all other drugs except Atorvastatin (Supplementary S10). Furthermore, forest plot results vs. control groups indicated that Spironolactone [MD = −5.00, 95% CI (−7.68, −2.32)] was the most effective intervention for reducing LVESD (Supplementary S7). Although the effect of Atorvastatin [MD = −1.96, 95% CI (−4.67, 0.75)] in reducing LVESD was less significant than that of Spironolactone, it still demonstrated relatively better efficacy in pairwise comparisons with Enalapril [MD = −3.96, 95% CI (−7.84, −0.08)] and Metoprolol [MD = −3.96, 95% CI (−7.74, −0.18)]. (Supplementary S7, S10) SUCRA results show that Spironolactone has the best effect on improving patients’ LVESD conditions: Spironolactone (99.2%) > Atorvastatin (81.0%) > Carvedilol (61.0%) > Control group (53.7%) > Panax ginseng (45.9%) > N-acetylcysteine (38.9%) > Nebivolol (37.2%) > Enalapril (16.6%) > Metoprolol(16.5%).

#### 3.5.2 LVEDD

Eight studies reported on LVEDD, and the results included eight interventions (Figure 5). The results of the network meta-analysis show that the differences between pairwise comparisons of the interventions are not statistically significant. SUCRA results show: Spironolactone (77.1%) > Nebivolol (72.8%) > N-acetylcysteine (61.2%) > Panax ginseng (55.3%) > Carvedilol(46.3%) > Control group (38.4%) > Metoprolol (30.4%) > Enalapril(18.7%).

#### 3.5.3 E/A

Eight studies reported on E/A, and the results included seven types of interventions (Figure 5). The analysis results indicate that Nebivolol performs remarkably well in improving the E/A ratio in patients. Compared with Carvedilol [MD = 0.20, 95% CI (0.01, 0.39)], Enalapril [MD = 0.23, 95% CI (0.06, 0.40)], and Telmisartan [MD = 0.42, 95% CI (0.16, 0.68)], its effects are significantly superior. Additionally, compared with Telmisartan, Metoprolol [MD = 0.29, 95% CI (0.02, 0.56)] also demonstrates better efficacy. The forest plot analysis further confirms these findings. Compared with the control group, Nebivolol significantly increases the E/A ratio [MD = 0.23, 95% CI (0.09, 0.37)], indicating its potential benefits in improving left ventricular diastolic function. In contrast, Telmisartan shows a trend of decreasing the E/A ratio [MD = −0.19, 95% CI (−0.41, 0.03)]. SUCRA results show: Nebivolol (94.9%) > Metoprolol (71.4%) > Carvedilol (52.1%) > N-acetylcysteine (45.4%) > Enalapril (40.2%) > Control group (39.6%) > Telmisartan (6.4%).

### 3.6 Inconsistency or heterogeneity/sensitive analysis

The publication bias test was conducted by drawing a comparison-corrected funnel plot of LVEF, LVEDD, LVESD, E/A, and cardiac events. Firstly, in terms of LVEF, the overall effect distribution is symmetrical, suggesting a low risk of publication bias; the majority of trial points are located to the right of the zero line, indicating that the intervention group generally significantly improved LVEF compared to the control group, and the concentrated distribution of effect values suggests that this indicator is sensitive to drug treatment and has strong repeatability. Secondly, for cardiac adverse events, the funnel plot shows good symmetry, but the effect values are more dispersed, suggesting that there may be some heterogeneity in the safety benefits of different interventions, and further expansion of the sample size is still needed to stabilize the estimates. The effect values for the E/A ratio are slightly skewed to the right around the zero line, and the confidence interval is slightly wide, suggesting that the intervention may have a limited effect on improving diastolic function, and the clinical benefits are still uncertain. The effect sizes of LVEDD and LVESD were concentrated in the 0-2 range, exhibiting a mild right skew, suggesting potential benefits of the intervention in terms of ventricular reverse remodeling. Overall, with the exception of the E/A ratio, the remaining four outcome measures demonstrated a general trend favoring the intervention over the control, and no significant publication bias was detected, supporting the credibility of the current evidence (Supplementary S9).

## 4 Discussion

Anthracycline chemotherapeutic agents play a crucial role in the treatment of various malignancies, including breast cancer and lymphoma. However, their associated cardiotoxicity has increasingly garnered clinical attention. Numerous studies have confirmed that anthracyclines can lead to a reduction in LVEF, which is closely related to the cumulative dose of the drug (20). systematically evaluated the impact of anthracycline chemotherapy on left ventricular remodeling using cardiac magnetic resonance imaging (CMR) technology. The study included 61 patients who received anthracycline chemotherapy, 15 patients who received non-anthracycline chemotherapy, and 24 healthy control participants without cancer. The results indicated that, in patients treated with anthracycline-based chemotherapy, LVEF significantly decreased by 5% within 6 months after treatment while no significant changes in LVEF were observed in patients treated with non-anthracycline-based chemotherapy or in the control group.This reduction in LVEF not only indicates impaired cardiac systolic function but is also significantly associated with the risk of adverse cardiovascular events, such as myocardial infarction and arrhythmias. A related prospective cohort study (21)further evaluated the relationship between LVEF and the risk of cardiovascular diseases, finding that when LVEF falls below 55%, the risk of heart failure significantly increases (HR = 1.15, 95% CI = 1.02–1.30).Left ventricular end-diastolic diameter (LVEDD) and end-systolic diameter (LVESD) are important indicators for assessing ventricular remodeling (22)., through the analysis of 15 randomized controlled trials, found that neurohormonal blockers may have cardioprotective effects. Among the studies, it was noted that patients treated with Nebivolol maintained stable LVEDD and LVESD after chemotherapy, whereas patients receiving placebo experienced an increase in LVEDD from 47.2 ± 3.8 mm to 52.0 ± 4.6 mm, and in LVESD from 29.7 ± 3.4 mm to 33.4 ± 4.5 mm. Additionally, changes in the diastolic function index, the E/A ratio, are also noteworthy. The systematic review and meta-analysis by (23) included 13 studies with a total of 892 breast cancer patients who underwent echocardiography both before and after anthracycline chemotherapy. The results showed a significant decrease in the E/A ratio in patients after anthracycline chemotherapy [MD = −0.14, 95% CI (−0.22, −0.06)], which may indicate early impairment of left ventricular diastolic function.

This systematic review and network meta-analysis synthesized evidence from 28 randomized controlled trials involving 2,858 patients, evaluating the comparative efficacy of 21 drug interventions for preventing anthracycline-induced cardiotoxicity across five outcome measures.The analysis revealed Spironolactone as the most effective intervention for improving both LVEF and LVESD, suggesting its potential advantages in maintaining or restoring cardiac function. This finding aligns with the network meta-analysis conducted by (24) and the cohort study by (25). This may be attributed to the fact that Spironolactone can effectively inhibit the apoptosis of cardiomyocyte during ischemia-reperfusion, reduce the occurrence of myocardial damage and fibrosis, promote the positive remodeling of cardiac structure and functional recovery while reducing sodium and water retention and lowering blood pressure, consequently alleviating the strain on the heart (26–28). Additionally, Lisinopril + Bisoprolol and Astragalus polysaccharide also demonstrated favorable improvements in LVEF, suggesting that multi-target interventions may offer greater cardioprotective potential compared to single-drug therapies. Notably, Panax ginseng exhibited paradoxical effects. Although it is significantly associated with a decrease in LVEF [OR = −6.90, 95% CI (−10.46, −3.34)], it ranks best in reducing cardiac events in the SUCRA analysis [OR = 0.07, 95% CI (0.00, 1.47)]. This contradiction may stem from heterogeneity among studies, insufficient sample size, or varying effects of ginseng on different cardiac function indicators. Panax ginseng may reduce myocardial injury events through improves antioxidant or myocardial tolerance, but it might have adverse effects on cardiac contractile function (LVEF), suggesting the need for larger-scale, more rigorously designed clinical studies to further validate the true impact of Panax ginseng on anthracycline-induced cardiotoxicity. Dexrazoxane and Vitamin E + Levocarnitine are more effective than conventional chemotherapy and placebo in reducing the incidence of cardiac events. As an iron chelator, Dexrazoxane can effectively prevent anthracycline-induced cardiotoxicity without compromising antineoplastic efficacy (29), and has been recommended by multiple guidelines for cardiac protection in anthracycline therapy. Similarly, carvedilol (both as monotherapy and in combination with candesartan) showed dual benefits in preserving LVEF while decreasing cardiac events, attributable to its pleiotropic effects including antioxidant, anti-inflammatory and anti-fibrotic properties (30). The superior performance of Nebivolol in improving E/A ratio is related to its characteristics as a beta-blocker, which can reduce heart rate and blood pressure, decrease cardiac load, and improve diastolic function (31, 32). In this study, the effects of various drug interventions on LVEDD were not significant, which may suggest that existing drug interventions still need further optimization and exploration for this indicator.

In recent years, Chinese herbal medicine has garnered significant attention in the prevention and treatment of cardiovascular diseases due to its multi-target pharmacological actions and favorable safety profile. Salidroside, a natural compound extracted from Rhodiola rosea, has demonstrated notable biological activities in various experimental models, including anti-tumor, antioxidant, anti-inflammatory, neuroprotective, and cardiovascular protective effects (33). It can mitigate anthracycline-induced myocardial injury by inhibiting oxidative stress and mitochondrial apoptosis through the activation of the Nr2/Keap-1/HO-1 and PI3K/Akt pathways (34). Astragalus polysaccharides, the most abundant and immunologically active components in Astragalus, can activate macrophages and T/B lymphocytes, upregulate the secretion of immune factors, and function as natural immune enhancers (35). The study further confirmed that Astragalus polysaccharides induce macrophage polarization toward the anti-inflammatory M2 phenotype by enhancing the Nrf2/HO-1 pathway, thereby improving vascular endothelial dysfunction in diabetic patients (36). Hong Huang decoction, a traditional Chinese medicine formula composed of various herbal ingredients including Astragalus and Salvia miltiorrhiza, exhibits synergistic antioxidant, anti-inflammatory, and microvascular dilation effects (37). This study found that the formula outperformed the control group in reducing overall cardiac events, suggesting its comprehensive effects may surpass those of single components. Rg3, one of the main active components of Panax ginseng, enhances cardiac function in a dose-dependent manner. Rg3 inhibits oxidative stress and apoptosis by downregulating miR-128-3p and upregulating the expression of double minute 4 protein (MDM4) (38). However, achieving efficacy comparable to conventional drugs requires higher doses, and its clinical application is still limited due to its low oral bioavailability (≈10%) and poor intestinal absorption (39, 40).

Compared to previous studies, this research has updated the latest findings and comprehensively evaluated the relative efficacy of various interventions using multiple objective outcome measures. However, certain limitations in the study design remain. Firstly, approximately half of the original studies were rated as having a high risk of bias, primarily due to insufficient description of blinding; given that all outcomes were objectively measured (e.g., laboratory indicators, incidence rates, etc.), the impact of the lack of blinding on the results may be limited, but it could still slightly overestimate the efficacy due to other potential biases (e.g., incomplete allocation concealment). Secondly, some studies had small sample sizes, which reduced the stability of the effect estimates. In the existing evidence network, comparisons of certain intervention pairs relied solely on direct evidence, lacking closed-loop indirect comparisons, which may limit precision. Future research should focus on conducting more high-quality, large-sample randomized controlled trials and further validate and consolidate the conclusions of this study through more comprehensive literature inclusion.

## 5 Conclusion

This systematic evaluation reveals significant heterogeneity in the cardioprotective efficacy of various pharmacological interventions against anthracycline-induced cardiotoxicity. Spironolactone demonstrates outstanding performance in improving LVEF and LVESD. Additionally, Lisinopril + Bisoprolol and Astragalus polysaccharides also show clinically meaningful improvements in LVEF, suggesting their value as alternative or adjunctive therapies. Dexrazoxane and Vitamin E + Levocarnitine demonstrate superior efficacy in reducing cardiac event rates compared to conventional chemotherapy regimens. Meanwhile, the treatment regimen of Carvedilol alone or in combination with Candesartan provides comprehensive protection by simultaneously preserving systolic function and reducing cardiac events. However, the effects of Panax ginseng on cardiac function present a paradox: while it is associated with a potential adverse effects on LVEF, it also exhibits a protective effect in reducing cardiac events. This paradox underscores the need for mechanistic studies to elucidate its complex pharmacodynamics and rigorously designed, large-scale clinical trials with standardized endpoints. For optimal patient management, we recommend that patients undergoing anthracycline chemotherapy, especially those at high risk of cardiotoxicity, should adopt individualized cardioprotective strategies. Clinical decision-making should comprehensively consider the patient's specific conditions, drug characteristics, and existing evidence-based medical data to achieve optimal cardioprotective outcomes. Through rational pharmacological interventions, the risk of cardiotoxicity can be effectively reduced, thereby improving patients' quality of life and prognosis.

## 6 Safety considerations for Key interventions

Although this analysis focuses on efficacy, the safety profile is equally important for clinical decision-making and efficacy evaluation.

Spironolactone is a potassium-sparing diuretic that may lead to hyperkalemia, especially in patients with renal insufficiency or those concurrently using other medications that increase potassium levels, such as ACE inhibitors (41). During its use, close monitoring of the patient's electrolyte balance, liver and kidney function, and endocrine system changes is essential.

Some studies suggest that prolonged or excessive use of Panax ginseng may lead to “ginseng abuse syndrome,” characterized by symptoms such as hypertension, insomnia, anxiety, and skin reactions (42). When used concurrently with anticoagulant drugs like warfarin, Panax ginseng may interfere with blood clotting and increase the risk of bleeding (43).

Dexrazoxane can significantly reduce the cardiotoxicity of anthracyclines, but its primary side effect is bone marrow suppression (reduction in white blood cells, neutrophils, and platelets). Additionally, long-term or high-dose use is associated with an increased risk of secondary malignancies and infertility (44).

Vitamin E is generally safe at conventional doses, but long-term or high-dose supplementation can inhibit the synthesis of vitamin K-dependent coagulation factors, reduce platelet aggregation, and thereby increase the risk of nosebleeds, hematuria, intraoperative and postoperative bleeding, and cerebral hemorrhage, especially when used in combination with anticoagulants such as warfarin and aspirin, or in individuals with pre-existing bleeding disorders (45).

The common side effects of Levocarnitine are mainly gastrointestinal discomfort (nausea, diarrhea, stomach cramps) and abnormal body odor; occasionally, dizziness, headache, or rash may occur; seizures and elevated blood pressure may occur with high doses or in patients with renal insufficiency.

The adverse reactions of Nebivolol are usually mild and occur less frequently than traditional beta-blockers (such as Atenolol, Metoprolol). Its nitric oxide(NO)-potentiating vasodilation may reduce some typical beta-blocker side effects (such as erectile dysfunction). However, liver dysfunction, bradycardia, and hypotension still require vigilance, especially in special populations (46).

## Data Availability

The original contributions presented in the study are included in the article/supplementary material, further inquiries can be directed to the corresponding author.
