# Supplementary material for: Pharmacological interventions to prevent cardiotoxicity in patients undergoing anthracycline-based chemotherapy: a network meta-analysis
Source: Front Cardiovasc Med. 2025 Sep 3;12:1612060. doi: 10.3389/fcvm.2025.1612060 (PMC12442320; doi:10.3389/fcvm.2025.1612060)
Supplement: Supplementary file 1 [file Datasheet1.docx]

**Supplement S1: Full search strategy.**

**Pubmed**

| **Anthracyclines** | |
| --- | --- |
| **Anthracyclines[Mesh] OR Anthracyclin*** |  |
| **"Aclarubicin"[Mesh] “Aclacinomycin A”“Aclaplastin”** | ((("Aclarubicin"[Mesh]) OR ((Aclacinomycin A) OR (Aclaplastin))) OR ((Aclacinomycin A[Title/Abstract]) OR (Aclaplastin[Title/Abstract]))) OR ((Aclacinomycin A[Text Word]) OR (Aclaplastin[Text Word])) |
| **"Daunorubicin"[Mesh] “Daunomycin”“Rubomycin”“Dauno Rubidomycine”“Rubidomycin”“Daunoblastin”“Daunoblastine”“Cerubidine”“Daunorubicin Hydrochloride”“Hydrochloride, Daunorubicin”** | ((("Daunorubicin"[Mesh]) OR (((((((((Daunomycin) OR (Rubomycin)) OR (Dauno Rubidomycine)) OR (Rubidomycin)) OR (Daunoblastin)) OR (Daunoblastine)) OR (Cerubidine)) OR (Daunorubicin Hydrochloride)) OR (Hydrochloride, Daunorubicin))) OR (((((((((Daunomycin[Title/Abstract]) OR (Rubomycin[Title/Abstract])) OR (Dauno Rubidomycine[Title/Abstract])) OR (Rubidomycin[Title/Abstract])) OR (Daunoblastin[Title/Abstract])) OR (Daunoblastine[Title/Abstract])) OR (Cerubidine[Title/Abstract])) OR (Daunorubicin Hydrochloride[Title/Abstract])) OR (Hydrochloride, Daunorubicin[Title/Abstract]))) OR (((((((((Daunomycin[Text Word]) OR (Rubomycin[Text Word])) OR (Dauno Rubidomycine[Text Word])) OR (Rubidomycin[Text Word])) OR (Daunoblastin[Text Word])) OR (Daunoblastine[Text Word])) OR (Cerubidine[Text Word])) OR (Daunorubicin Hydrochloride[Text Word])) OR (Hydrochloride, Daunorubicin[Text Word])) |
| **"Carubicin"[Mesh] "Carminomycin" "Demethyldaunorubicin" "Karminomycin" "Carminomicin" "Carubicin Hydrochloride" "Hydrochloride, Carubicin"** | (((Carubicin[Mesh]) OR ((((((Carminomycin) OR (Demethyldaunorubicin)) OR (Karminomycin)) OR (Carminomicin)) OR (Carubicin Hydrochloride)) OR (Hydrochloride, Carubicin))) OR ((((((Carminomycin[Title/Abstract]) OR (Demethyldaunorubicin[Title/Abstract])) OR (Karminomycin[Title/Abstract])) OR (Carminomicin[Title/Abstract])) OR (Carubicin Hydrochloride[Title/Abstract])) OR (Hydrochloride, Carubicin[Title/Abstract]))) OR ((((((Carminomycin[Text Word]) OR (Demethyldaunorubicin[Text Word])) OR (Karminomycin[Text Word])) OR (Carminomicin[Text Word])) OR (Carubicin Hydrochloride[Text Word])) OR (Hydrochloride, Carubicin[Text Word])) |
| **Doxorubicin[Mesh] "Rubex" "Adriamycin" "Adriblastin" "Adriblastine" "Adriblastina" "Adriablastine" "Adriablastin" "Myocet"** | (((Doxorubicin[Mesh]) OR ((((((((Rubex) OR (Adriamycin)) OR (Adriblastin)) OR (Adriblastine)) OR (Adriblastina)) OR (Adriablastine)) OR (Adriablastin)) OR (Myocet))) OR ((((((((Rubex[Title/Abstract]) OR (Adriamycin[Title/Abstract])) OR (Adriblastin[Title/Abstract])) OR (Adriblastine[Title/Abstract])) OR (Adriblastina[Title/Abstract])) OR (Adriablastine[Title/Abstract])) OR (Adriablastin[Title/Abstract])) OR (Myocet[Title/Abstract]))) OR ((((((((Rubex[Text Word]) OR (Adriamycin[Text Word])) OR (Adriblastin[Text Word])) OR (Adriblastine[Text Word])) OR (Adriblastina[Text Word])) OR (Adriablastine[Text Word])) OR (Adriablastin[Text Word])) OR (Myocet[Text Word])) |
| **Epirubicin[Mesh] "Ellence" "Pharmorubicin" "Farmorubicine" "Farmorubicin" "Epirubicin Hydrochloride" "Hydrochloride, Epirubicin"** | (((Epirubicin[Mesh]) OR ((((((Ellence) OR (Pharmorubicin)) OR (Farmorubicine)) OR (Farmorubicin)) OR (Epirubicin Hydrochloride)) OR (Hydrochloride, Epirubicin))) OR ((((((Ellence[Title/Abstract]) OR (Pharmorubicin[Title/Abstract])) OR (Farmorubicine[Title/Abstract])) OR (Farmorubicin[Title/Abstract])) OR (Epirubicin Hydrochloride[Title/Abstract])) OR (Hydrochloride, Epirubicin[Title/Abstract]))) OR ((((((Ellence[Text Word]) OR (Pharmorubicin[Text Word])) OR (Farmorubicine[Text Word])) OR (Farmorubicin[Text Word])) OR (Epirubicin Hydrochloride[Text Word])) OR (Hydrochloride, Epirubicin[Text Word])) |
| **Idarubicin[Mesh]) "Idarubicin Hydrochloride" "Hydrochloride, Idarubicin"** | (((Idarubicin[Mesh]) OR ((Idarubicin Hydrochloride) OR (Hydrochloride, Idarubicin))) OR (((Idarubicin Hydrochloride[Title/Abstract])) OR (Hydrochloride, Idarubicin[Title/Abstract]))) OR (((Idarubicin Hydrochloride[Text Word])) OR (Hydrochloride, Idarubicin[Text Word])) |
| **Nogalamycin[Mesh]** |  |
| **Menogaril[Mesh]** |  |
| **Cardiotoxicity** | |
| **Cardiotoxicity[Mesh] "Cardiotoxicities" "Cardiac Toxicity" "Cardiac Toxicities" "Toxicity, Cardiac"** | (Cardiotoxicity[Mesh]) OR ((((((Cardiotoxicities) OR (Cardiac Toxicity)) OR (Cardiac Toxicities)) OR (Toxicity, Cardiac)) OR ((((Cardiotoxicities[Title/Abstract]) OR (Cardiac Toxicity[Title/Abstract])) OR (Cardiac Toxicities[Title/Abstract])) OR (Toxicity, Cardiac[Title/Abstract]))) OR ((((Cardiotoxicities[Text Word]) OR (Cardiac Toxicity[Text Word])) OR (Cardiac Toxicities[Text Word])) OR (Toxicity, Cardiac[Text Word]))) |
| **Heart Diseases[Mesh] "heart diseas*" "cardiac diseas*"** | (((Heart Diseases[Mesh]) OR ((heart diseas*) OR (cardiac diseas*))) OR ((heart diseas*[Title/Abstract]) OR (cardiac diseas*[Title/Abstract]))) OR ((heart diseas*[Text Word]) OR (cardiac diseas*[Text Word])) |
| **Arrhythmias, Cardiac** | ((Arrhythmias, Cardiac) OR (Arrhythmias, Cardiac[Title/Abstract])) OR (Arrhythmias, Cardiac[Text Word]) |
| **Cardiomyopathies** | ((Cardiomyopathies) OR (Cardiomyopathies[Title/Abstract])) OR (Cardiomyopathies[Text Word]) |
| **Heart Failure** | ((Heart Failure) OR (Heart Failure[Title/Abstract])) OR (Heart Failure[Text Word]) |
| **Coronary Disease +** | ((Coronary Disease +) OR (Coronary Disease +[Title/Abstract])) OR (Coronary Disease +[Text Word]) |
| **Myocardial Infarction +** | ((Ventricular Dysfunction) OR (Ventricular Dysfunction[Title/Abstract])) OR (Ventricular Dysfunction[Text Word]) |
| **"Treatment" "Prevention" "chemotherapy"** | ((Treatment) OR (Prevention)) OR (chemotherapy) |
| Results | 4,957 |

**Embase**

| **Anthracyclines** | |
| --- | --- |
| **'Anthracyclines' OR 'Anthracyclin*'** | 'anthracyclines'/exp OR 'anthracyclines' OR 'anthracyclin*' OR anthracyclin* OR anthracyclin*:ti,ab,kw |
| **'Aclarubicin' OR 'Aclacinomycin A' OR 'Aclaplastin'** | 'aclarubicin'/exp OR 'aclarubicin' OR 'aclacinomycin a'/exp OR 'aclaplastin'/exp OR 'aclacinomycin a' OR 'aclaplastin' OR 'aclacinomycin a':ti,ab,kw OR 'aclaplastin':ti,ab,kw |
| **‘Daunorubicin’ OR ‘Daunomycin’ OR ‘Rubomycin’ OR ‘Dauno Rubidomycine’ OR ‘Rubidomycin’ OR ‘Daunoblastin’ OR ‘Daunoblastine’ OR ‘Cerubidine’ ‘Daunorubicin Hydrochloride’ OR ‘Hydrochloride, Daunorubicin’** | ('daunorubicin'/exp OR 'daunorubicin' OR 'daunomycin'/exp OR 'daunomycin' OR 'rubomycin'/exp OR 'rubomycin' OR 'dauno rubidomycine' OR 'rubidomycin'/exp OR 'rubidomycin' OR 'daunoblastin'/exp OR 'daunoblastin' OR 'daunoblastine'/exp OR 'daunoblastine' OR 'cerubidine'/exp OR 'cerubidine') AND ('daunorubicin hydrochloride'/exp OR 'daunorubicin hydrochloride') OR 'daunorubicin' OR 'daunomycin' OR 'rubomycin' OR 'dauno rubidomycine' OR 'rubidomycin' OR 'daunoblastin' OR 'daunoblastine' OR 'cerubidine' OR 'daunorubicin hydrochloride' OR 'hydrochloride, daunorubicin' OR 'daunorubicin':ti,ab,kw OR 'daunomycin':ti,ab,kw OR 'rubomycin':ti,ab,kw OR 'dauno rubidomycine':ti,ab,kw OR 'rubidomycin':ti,ab,kw OR 'daunoblastin':ti,ab,kw OR 'daunoblastine':ti,ab,kw OR 'cerubidine':ti,ab,kw OR 'daunorubicin hydrochloride':ti,ab,kw OR 'hydrochloride, daunorubicin':ti,ab,kw |
| **‘Carubicin’ OR ‘Carminomycin’ OR ‘Demethyldaunorubicin’ OR ‘Karminomycin’ OR ‘Carminomicin’ OR ‘Carubicin Hydrochloride’ OR ‘Hydrochloride, Carubicin’** | 'carubicin'/exp OR 'carubicin' OR 'carminomycin'/exp OR 'karminomycin'/exp OR 'carminomycin' OR 'demethyldaunorubicin' OR 'karminomycin' OR 'carminomicin' OR 'carubicin hydrochloride' OR 'hydrochloride, carubicin' OR 'carminomycin':ti,ab,kw OR 'demethyldaunorubicin':ti,ab,kw OR 'karminomycin':ti,ab,kw OR 'carminomicin':ti,ab,kw OR 'carubicin hydrochloride':ti,ab,kw OR 'hydrochloride, carubicin':ti,ab,kw |
| **'Doxorubicin' OR 'Rubex' OR 'Adriamycin' OR 'Adriblastin' OR 'Adriblastine' OR 'Adriblastina' OR 'Adriablastine' OR 'Adriablastin' OR 'Myocet'** | 'doxorubicin'/exp OR 'rubex'/exp OR 'adriamycin'/exp OR 'adriblastin'/exp OR 'adriblastine'/exp OR 'adriblastina'/exp OR 'adriablastine'/exp OR 'adriablastin'/exp OR 'myocet'/exp OR 'doxorubicin' OR 'rubex' OR 'adriamycin' OR 'adriblastin' OR 'adriblastine' OR 'adriblastina' OR 'adriablastine' OR 'adriablastin' OR 'myocet' OR 'doxorubicin':ti,ab,kw OR 'rubex':ti,ab,kw OR 'adriamycin':ti,ab,kw OR 'adriblastin':ti,ab,kw OR 'adriblastine':ti,ab,kw OR 'adriblastina':ti,ab,kw OR 'adriablastine':ti,ab,kw OR 'adriablastin':ti,ab,kw OR 'myocet':ti,ab,kw |
| **'Epirubicin' OR 'Ellence' OR 'Pharmorubicin' OR 'Farmorubicine' OR 'Farmorubicin' OR 'Epirubicin Hydrochloride' OR 'Hydrochloride, Epirubicin'** | 'epirubicin'/exp OR 'epirubicin' OR 'ellence'/exp OR 'pharmorubicin'/exp OR 'farmorubicine'/exp OR 'farmorubicin'/exp OR 'epirubicin hydrochloride'/exp OR 'ellence' OR 'pharmorubicin' OR 'farmorubicine' OR 'farmorubicin' OR 'epirubicin hydrochloride' OR 'hydrochloride, epirubicin' OR 'ellence':ti,ab,kw OR 'pharmorubicin':ti,ab,kw OR 'farmorubicine':ti,ab,kw OR 'farmorubicin':ti,ab,kw OR 'epirubicin hydrochloride':ti,ab,kw OR 'hydrochloride, epirubicin':ti,ab,kw |
| **'Idarubicin' OR 'Idarubicin Hydrochloride' OR 'Hydrochloride, Idarubicin'** | 'idarubicin'/exp OR 'idarubicin' OR 'idarubicin hydrochloride'/exp OR 'idarubicin hydrochloride' OR 'hydrochloride, idarubicin' OR 'idarubicin hydrochloride':ti,ab,kw OR 'hydrochloride, idarubicin':ti,ab,kw |
| **Nogalamycin** | 'nogalamycin'/exp OR nogalamycin OR nogalamycin:ti,ab,kw |
| **Menogaril** | 'menogaril'/exp OR menogaril OR menogaril:ti,ab,kw |
| **Cardiotoxicity** | |
| **‘Cardiotoxicity’ OR ‘Cardiotoxicities’ OR ‘Cardiac Toxicity’ OR ‘Cardiac Toxicities’ OR ‘Toxicity, Cardiac** | 'cardiotoxicity'/exp OR 'cardiotoxicity' OR 'cardiac toxicity'/exp OR 'cardiotoxicities' OR 'cardiac toxicity' OR 'cardiac toxicities' OR 'toxicity, cardiac' OR 'cardiotoxicities':ti,ab,kw OR 'cardiac toxicity':ti,ab,kw OR 'cardiac toxicities':ti,ab,kw OR 'toxicity, cardiac':ti,ab,kw |
| **‘Heart Diseases’ OR ‘heart diseas*’ OR ‘cardiac diseas*’** | 'heart diseases'/exp OR 'heart diseases' OR 'heart diseas*' OR 'cardiac diseas*' OR 'heart diseas*':ti,ab,kw OR 'cardiac diseas*':ti,ab,kw |
| **Arrhythmias, Cardiac** | 'arrhythmias, cardiac'/exp OR 'arrhythmias, cardiac' OR (arrhythmias, AND ('cardiac'/exp OR cardiac)) |
| **Cardiomyopathies** | 'cardiomyopathies'/exp OR cardiomyopathies |
| **Heart Failure** | 'heart failure'/exp OR 'heart failure' OR (('heart'/exp OR heart) AND ('failure'/exp OR failure)) |
| **Coronary Disease +** | 'coronary disease +'/exp OR 'coronary disease +' OR (coronary AND ('disease'/exp OR disease) AND +) |
| **Myocardial Infarction +** | 'myocardial infarction +'/exp OR 'myocardial infarction +' OR (myocardial AND ('infarction'/exp OR infarction) AND +) |
| **Ventricular Dysfunction** | 'ventricular dysfunction'/exp OR 'ventricular dysfunction' OR (ventricular AND dysfunction) |
| **‘Treatment’ OR ‘Prevention’ OR ‘chemotherapy’** | 'treatment'/exp OR treatment OR 'prevention'/exp OR prevention OR 'chemotherapy'/exp OR chemotherapy |
| Results | 9,066 |

**Wecb of Science**

| **Anthracyclines** | |
| --- | --- |
| **'Anthracyclines' OR 'Anthracyclin*'** | (ALL=(Anthracyclines)) OR ALL=(Anthracyclin*)  (TS=(Anthracyclines)) OR TS=(Anthracyclin*)  (TI=(Anthracyclines)) OR TI=(Anthracyclin*)  (AB=(Anthracyclines)) OR AB=(Anthracyclin*)  (AK=(Anthracyclines)) OR AK=(Anthracyclin*) |
| **'Aclarubicin' OR 'Aclacinomycin A' OR 'Aclaplastin'** | ((ALL=(Aclarubicin)) OR ALL=(Aclacinomycin)) OR ALL=(Aclaplastin)  ((TS=(Aclarubicin)) OR TS=(Aclacinomycin)) OR TS=(Aclaplastin)  ((TI=(Aclarubicin)) OR TI=(Aclacinomycin)) OR TI=(Aclaplastin)  ((AB=(Aclarubicin)) OR AB=(Aclacinomycin)) OR AB=(Aclaplastin)  ((AK=(Aclarubicin)) OR AK=(Aclacinomycin)) OR AK=(Aclaplastin) |
| **‘Daunorubicin’ OR ‘Daunomycin’ OR ‘Rubomycin’ OR ‘Dauno Rubidomycine’ OR ‘Rubidomycin’ OR ‘Daunoblastin’ OR ‘Daunoblastine’ OR ‘Cerubidine’ ‘Daunorubicin Hydrochloride’** | ((((((((ALL=(Daunorubicin)) OR ALL=(Daunomycin)) OR ALL=(Rubomycin)) OR ALL=(Dauno Rubidomycine)) OR ALL=(Rubidomycin)) OR ALL=(Daunoblastin)) OR ALL=(Daunoblastine)) OR ALL=(Cerubidine)) OR ALL=(Daunorubicin Hydrochloride)  ((((((((TS=(Daunorubicin)) OR TS=(Daunomycin)) OR TS=(Rubomycin)) OR TS=(Dauno Rubidomycine)) OR TS=(Rubidomycin)) OR TS=(Daunoblastin)) OR TS=(Daunoblastine)) OR TS=(Cerubidine)) OR TS=(Daunorubicin Hydrochloride)  ((((((((TI=(Daunorubicin)) OR TI=(Daunomycin)) OR TI=(Rubomycin)) OR TI=(Dauno Rubidomycine)) OR TI=(Rubidomycin)) OR TI=(Daunoblastin)) OR TI=(Daunoblastine)) OR TI=(Cerubidine)) OR TI=(Daunorubicin Hydrochloride)  ((((((((AB=(Daunorubicin)) OR AB=(Daunomycin)) OR AB=(Rubomycin)) OR AB=(Dauno Rubidomycine)) OR AB=(Rubidomycin)) OR AB=(Daunoblastin)) OR AB=(Daunoblastine)) OR AB=(Cerubidine)) OR AB=(Daunorubicin Hydrochloride)  ((((((((AK=(Daunorubicin)) OR AK=(Daunomycin)) OR AK=(Rubomycin)) OR AK=(Dauno Rubidomycine)) OR AK=(Rubidomycin)) OR AK=(Daunoblastin)) OR AK=(Daunoblastine)) OR AK=(Cerubidine)) OR AK=(Daunorubicin Hydrochloride) |
| **‘Carubicin’ OR ‘Carminomycin’ OR ‘Demethyldaunorubicin’ OR ‘Karminomycin’ OR ‘Carminomicin’ OR ‘Carubicin Hydrochloride’** | (((((ALL=(Carubicin)) OR ALL=(Carminomycin)) OR ALL=(Demethyldaunorubicin)) OR ALL=(Karminomycin)) OR ALL=(Carminomicin)) OR ALL=(Carubicin Hydrochloride)  (((((TS=(Carubicin)) OR TS=(Carminomycin)) OR TS=(Demethyldaunorubicin)) OR TS=(Karminomycin)) OR TS=(Carminomicin)) OR TS=(Carubicin Hydrochloride)  (((((TI=(Carubicin)) OR TI=(Carminomycin)) OR TI=(Demethyldaunorubicin)) OR TI=(Karminomycin)) OR TI=(Carminomicin)) OR TI=(Carubicin Hydrochloride)  (((((AB=(Carubicin)) OR AB=(Carminomycin)) OR AB=(Demethyldaunorubicin)) OR AB=(Karminomycin)) OR AB=(Carminomicin)) OR AB=(Carubicin Hydrochloride)  (((((AK=(Carubicin)) OR AK=(Carminomycin)) OR AK=(Demethyldaunorubicin)) OR AK=(Karminomycin)) OR AK=(Carminomicin)) OR AK=(Carubicin Hydrochloride) |
| **'Doxorubicin' OR 'Rubex' OR 'Adriamycin' OR 'Adriblastin' OR 'Adriblastine' OR 'Adriblastina' OR 'Adriablastine' OR 'Adriablastin' OR 'Myocet'** | ((((((((ALL=(Doxorubicin)) OR ALL=(Rubex)) OR ALL=(Adriamycin)) OR ALL=(Adriblastin)) OR ALL=(Adriblastine)) OR ALL=(Adriblastina)) OR ALL=(Adriablastine)) OR ALL=(Adriablastin)) OR ALL=(Myocet)  ((((((((TS=(Doxorubicin)) OR TS=(Rubex)) OR TS=(Adriamycin)) OR TS=(Adriblastin)) OR TS=(Adriblastine)) OR TS=(Adriblastina)) OR TS=(Adriablastine)) OR TS=(Adriablastin)) OR TS=(Myocet)  ((((((((TI=(Doxorubicin)) OR TI=(Rubex)) OR TI=(Adriamycin)) OR TI=(Adriblastin)) OR TI=(Adriblastine)) OR TI=(Adriblastina)) OR TI=(Adriablastine)) OR TI=(Adriablastin)) OR TI=(Myocet)  ((((((((AB=(Doxorubicin)) OR AB=(Rubex)) OR AB=(Adriamycin)) OR AB=(Adriblastin)) OR AB=(Adriblastine)) OR AB=(Adriblastina)) OR AB=(Adriablastine)) OR AB=(Adriablastin)) OR AB=(Myocet)  ((((((((AK=(Doxorubicin)) OR AK=(Rubex)) OR AK=(Adriamycin)) OR AK=(Adriblastin)) OR AK=(Adriblastine)) OR AK=(Adriblastina)) OR AK=(Adriablastine)) OR AK=(Adriablastin)) OR AK=(Myocet) |
| **'Epirubicin' OR 'Ellence' OR 'Pharmorubicin' OR 'Farmorubicine' OR 'Farmorubicin' OR 'Epirubicin Hydrochloride'** | (((((ALL=(Epirubicin)) OR ALL=(Ellence)) OR ALL=(Pharmorubicin)) OR ALL=(Farmorubicine)) OR ALL=(Farmorubicin)) OR ALL=(Epirubicin Hydrochloride)  (((((TS=(Epirubicin)) OR TS=(Ellence)) OR TS=(Pharmorubicin)) OR TS=(Farmorubicine)) OR TS=(Farmorubicin)) OR TS=(Epirubicin Hydrochloride)  (((((TI=(Epirubicin)) OR TI=(Ellence)) OR TI=(Pharmorubicin)) OR TI=(Farmorubicine)) OR TI=(Farmorubicin)) OR TI=(Epirubicin Hydrochloride)  (((((AB=(Epirubicin)) OR AB=(Ellence)) OR AB=(Pharmorubicin)) OR AB=(Farmorubicine)) OR AB=(Farmorubicin)) OR AB=(Epirubicin Hydrochloride)  (((((AK=(Epirubicin)) OR AK=(Ellence)) OR AK=(Pharmorubicin)) OR AK=(Farmorubicine)) OR AK=(Farmorubicin)) OR AK=(Epirubicin Hydrochloride) |
| **'Idarubicin' OR 'Idarubicin Hydrochloride'** | (ALL=(Idarubicin)) OR ALL=(Idarubicin Hydrochloride)  (TI=(Idarubicin)) OR TI=(Idarubicin Hydrochloride)  (TS=(Idarubicin)) OR TS=(Idarubicin Hydrochloride)  (AB=(Idarubicin)) OR AB=(Idarubicin Hydrochloride)  (AK=(Idarubicin)) OR AK=(Idarubicin Hydrochloride) |
| **Nogalamycin** | ((((ALL=(Nogalamycin)) OR TS=(Nogalamycin)) OR TI=(Nogalamycin)) OR AB=(Nogalamycin)) OR AK=(Nogalamycin) |
| **Menogaril** | ((((ALL=(Menogaril)) OR TS=(Menogaril)) OR TI=(Menogaril)) OR AB=(Menogaril)) OR AK=(Menogaril) |
| **Cardiotoxicity** | |
| **‘Cardiotoxicity’ OR ‘Cardiotoxicities’ OR ‘Cardiac Toxicity’ OR ‘Cardiac Toxicities’** | (((ALL=(Cardiotoxicity)) OR ALL=(Cardiotoxicities)) OR ALL=(Cardiac Toxicity)) OR ALL=(Cardiac Toxicities)  (((TS=(Cardiotoxicity)) OR TS=(Cardiotoxicities)) OR TS=(Cardiac Toxicity)) OR TS=(Cardiac Toxicities)  (((TI=(Cardiotoxicity)) OR TI=(Cardiotoxicities)) OR TI=(Cardiac Toxicity)) OR TI=(Cardiac Toxicities)  (((AB=(Cardiotoxicity)) OR AB=(Cardiotoxicities)) OR AB=(Cardiac Toxicity)) OR AB=(Cardiac Toxicities)  (((AK=(Cardiotoxicity)) OR AK=(Cardiotoxicities)) OR AK=(Cardiac Toxicity)) OR AK=(Cardiac Toxicities) |
| **‘Heart Diseases’ OR ‘heart diseas*’ OR ‘cardiac diseas*’** | ((ALL=(Heart Diseases)) OR ALL=(heart diseas*)) OR ALL=(cardiac diseas*)  ((TS=(Heart Diseases)) OR TS=(heart diseas*)) OR TS=(cardiac diseas*)  ((TI=(Heart Diseases)) OR TI=(heart diseas*)) OR TI=(cardiac diseas*)  ((AB=(Heart Diseases)) OR AB=(heart diseas*)) OR AB=(cardiac diseas*)  ((AK=(Heart Diseases)) OR AK=(heart diseas*)) OR AK=(cardiac diseas*) |
| **Arrhythmias** | ((((ALL=(Arrhythmias)) OR TS=(Arrhythmias)) OR TI=(Arrhythmias)) OR AB=(Arrhythmias)) OR AK=(Arrhythmias) |
| **Cardiomyopathies** | ((((ALL=(Cardiomyopathies)) OR TS=(Cardiomyopathies)) OR TI=(Cardiomyopathies)) OR AB=(Cardiomyopathies)) OR AK=(Cardiomyopathies) |
| **Heart Failure** | ((((ALL=(Heart Failure)) OR TS=(Heart Failure)) OR TI=(Heart Failure)) OR AB=(Heart Failure)) OR AK=(Heart Failure) |
| **Coronary Disease +** | ((((ALL=(Coronary Disease +)) OR TS=(Coronary Disease +)) OR TI=(Coronary Disease +)) OR AB=(Coronary Disease +)) OR AK=(Coronary Disease +) |
| **Myocardial Infarction +** | ((((ALL=(Myocardial Infarction +)) OR TS=(Myocardial Infarction +)) OR TI=(Myocardial Infarction +)) OR AB=(Myocardial Infarction +)) OR AK=(Myocardial Infarction +) |
| **Ventricular Dysfunction** | ((((ALL=(Ventricular Dysfunction)) OR TS=(Ventricular Dysfunction)) OR TI=(Ventricular Dysfunction)) OR AB=(Ventricular Dysfunction)) OR AK=(Ventricular Dysfunction) |
| **‘Treatment’ OR ‘Prevention’ OR ‘chemotherapy’** | ((((ALL=(Treatment)) OR TS=(Treatment)) OR TI=(Treatment)) OR AB=(Treatment)) OR AK=(Treatment)  ((((ALL=(Prevention)) OR TS=(Prevention)) OR TI=(Prevention)) OR AB=(Prevention)) OR AK=(Prevention)  ((((ALL=(chemotherapy)) OR TS=(chemotherapy)) OR TI=(chemotherapy)) OR AB=(chemotherapy)) OR AK=(chemotherapy) |
| Results | 17,157 |

**Scopus**

**Anthracyclines**

'Anthracyclines' OR 'Anthracyclin*'

'Aclarubicin' OR 'Aclacinomycin A' OR 'Aclaplastin'

'Daunorubicin' OR 'Daunomycin' OR 'Rubomycin' OR 'Dauno Rubidomycine' OR 'Rubidomycin' OR 'Daunoblastin' OR 'Daunoblastine' OR 'Cerubidine' 'Daunorubicin Hydrochloride'

'Carubicin' OR 'Carminomycin' OR 'Demethyldaunorubicin' OR 'Karminomycin' OR 'Carminomicin' OR 'Carubicin Hydrochloride'

'Doxorubicin' OR 'Rubex' OR 'Adriamycin' OR 'Adriblastin' OR 'Adriblastine' OR 'Adriblastina' OR 'Adriablastine' OR 'Adriablastin' OR 'Myocet'

'Epirubicin' OR 'Ellence' OR 'Pharmorubicin' OR 'Farmorubicine' OR 'Farmorubicin' OR 'Epirubicin Hydrochloride'

'Idarubicin' OR 'Idarubicin Hydrochloride'

'Nogalamycin'

'Menogaril'

**Cardiotoxicity**

'Cardiotoxicity' OR 'Cardiotoxicities' OR 'Cardiac Toxicity' OR 'Cardiac Toxicities'

Heart Diseases OR heart diseas* OR cardiac diseas*

'Arrhythmias'

'Cardiomyopathies'

'Heart Failure'

'Coronary Disease'

'Myocardial Infarction'

'Ventricular Dysfunction'

'Treatment' OR 'Prevention' OR 'chemotherapy'

Results:13,922

**Cochrane Library**

**Anthracyclines**

Anthracyclines[Mesh] OR Anthracyclin*

Aclarubicin[Mesh] Aclacinomycin OR Aclaplastin

Daunorubicin[Mesh] Daunomycin OR Rubomycin OR Dauno Rubidomycine OR Rubidomycin OR Daunoblastin OR Daunoblastine OR Cerubidine OR Daunorubicin Hydrochloride

Carubicin[Mesh] Carminomycin OR Demethyldaunorubicin OR Karminomycin OR Carminomicin OR Carubicin Hydrochloride

Doxorubicin[Mesh] Rubex OR Adriamycin OR Adriblastin OR Adriblastine OR Adriblastina OR Adriablastine OR Adriablastin OR Myocet

Epirubicin[Mesh] Ellence OR Pharmorubicin OR Farmorubicine OR Farmorubicin OR Epirubicin

**Hydrochloride**

Idarubicin[Mesh] Idarubicin Hydrochloride

Nogalamycin[Mesh]

Menogaril[Mesh]

**Cardiotoxicity**

Cardiotoxicity[Mesh] Cardiotoxicities OR Cardiac Toxicity OR Cardiac Toxicities

Heart Diseases[Mesh] heart diseas* OR cardiac diseas*

Arrhythmias, Cardiac[Mesh]

Cardiomyopathies[Mesh]

Heart Failure[Mesh]

Coronary Disease [Mesh]

Myocardial Infarction [Mesh]

Ventricular Dysfunction[Mesh]

Treatment OR Prevention OR chemother

Results:9,752

**Supplement S2: Full reference list of referable studies**

1. Moustafa I, Connolly C, Anis M, Mustafa H, et al. A prospective study to evaluate the efficacy and safety of vitamin E and levocarnitine prophylaxis against doxorubicin-induced cardiotoxicity in adult breast cancer patients. *J Oncol Pharm Pract*. 2024;30(2):354-366. doi:10.1177/10781552231171114

2. Swain SM, Whaley FS, Gerber MC, et al. Cardioprotection with dexrazoxane for doxorubicin-containing therapy in advanced breast cancer. *JCO*. 1997;15(4):1318-1332. doi:10.1200/JCO.1997.15.4.1318

3. Georgakopoulos P, Roussou P, Matsakas E, et al. Cardioprotective effect of metoprolol and enalapril in doxorubicin‐treated lymphoma patients: A prospective, parallel‐group, randomized, controlled study with 36‐month follow‐up. *American J Hematol*. 2010;85(11):894-896. doi:10.1002/ajh.21840

4. Beheshti A, Mostafavi Toroghi H, Hosseini G, Zarifian A, Homaei Shandiz F, Fazlinezhad A. Carvedilol Administration Can Prevent Doxorubicin-Induced Cardiotoxicity: A Double-Blind Randomized Trial. *Cardiology*. 2016;134(1):47-53. doi:10.1159/000442722

5. Avila MS, Ayub-Ferreira SM, De Barros Wanderley MR, et al. Carvedilol for Prevention of Chemotherapy-Related Cardiotoxicity. *Journal of the American College of Cardiology*. 2018;71(20):2281-2290. doi:10.1016/j.jacc.2018.02.049

6. Cao S, Xue J, Chen L, et al. E ects of the Chinese herbal medicine Hong Huang decoction, on myocardial injury in breast cancer patients who underwent anthracycline-based chemotherapy. *Frontiers in Cardiovascular Medicine*. Published online 2022.

7. Elitok A, Oz F, Cizgici AY, et al. Effect of carvedilol on silent anthracycline-induced cardiotoxicity assessed by strain imaging: A prospective randomized controlled study with six-month follow-up. *Cardiol J*. 2014;21(5):509-515. doi:10.5603/CJ.a2013.0150

8. Janbabai G, Nabati M, Faghihinia M, Azizi S, Borhani S, Yazdani J. Effect of Enalapril on Preventing Anthracycline-Induced Cardiomyopathy. *Cardiovasc Toxicol*. 2017;17(2):130-139. doi:10.1007/s12012-016-9365-z

9. Jo SH, Kim LS, Kim SA, et al. Evaluation of Short-Term Use of N-Acetylcysteine as a Strategy for Prevention of Anthracycline-Induced Cardiomyopathy: EPOCH Trial - A Prospective Randomized Study. *Korean Circ J*. 2013;43(3):174. doi:10.4070/kcj.2013.43.3.174

10. Čiburienė E, Aidietienė S, Ščerbickaitė G, et al. Ivabradine for the Prevention of Anthracycline-Induced Cardiotoxicity in Female Patients with Primarily Breast Cancer: A Prospective, Randomized, Open-Label Clinical Trial. *Medicina*. 2023;59(12):2140. doi:10.3390/medicina59122140

11. Mantovani, G. M, Piras A, Madeddu C, et al. Long-term protective effects of the angiotensin receptor blocker telmisartan on epirubicin-induced inflammation, oxidative stress and myocardial dysfunction. *Experimental and Therapeutic Medicine*. 2011;2(5):1003-1009. doi:10.3892/etm.2011.305

12. Venturini M, Michelotti A, Del Mastro L, et al. Multicenter randomized controlled clinical trial to evaluate cardioprotection of dexrazoxane versus no cardioprotection in women receiving epirubicin chemotherapy for advanced breast cancer. *JCO*. 1996;14(12):3112-3120. doi:10.1200/JCO.1996.14.12.3112

13. Marty M, Espié M, Llombart A, Monnier A, Rapoport BL, Stahalova V. Multicenter randomized phase III study of the cardioprotective effect of dexrazoxane (Cardioxane®) in advanced/metastatic breast cancer patients treated with anthracycline-based chemotherapy. *Annals of Oncology*. 2006;17(4):614-622. doi:10.1093/annonc/mdj134

14. Henriksen PA, Hall P, MacPherson IR, et al. Multicenter, Prospective, Randomized Controlled Trial of High-Sensitivity Cardiac Troponin I–Guided Combination Angiotensin Receptor Blockade and Beta-Blocker Therapy to Prevent Anthracycline Cardiotoxicity: The Cardiac CARE Trial. *Circulation*. 2023;148(21):1680-1690. doi:10.1161/CIRCULATIONAHA.123.064274

15. Cochera F, Dinca D, Bordejevic DA, et al. Nebivolol effect on doxorubicin-induced cardiotoxicity in breast cancer. *CMAR*. 2018;Volume 10:2071-2081. doi:10.2147/CMAR.S166481

16. Hao W, Shi Y, Qin Y, et al. *Platycodon grandiflorum* Protects Against Anthracycline-Induced Cardiotoxicity in Early Breast Cancer Patients. *Integr Cancer Ther*. 2020;19:153473542094501. doi:10.1177/1534735420945017

17. Li X, Guo X, Li J, Yuan L, Wang H. Preventing effect of astragalus polysaccharide on cardiotoxicity induced by chemotherapy of epirubicin: A pilot study. *Medicine*. 2022;101(32):e30000. doi:10.1097/MD.0000000000030000

18. Milei J, Marantz A, Alé J, Vazquez A, Buceta JE. Prevention of Adriamycin-Induced Cardiotoxicity by Prenylamine: A Pilot Double Blind Study. *Cancer Drug Delivery*. 1987;4(2):129-136. doi:10.1089/cdd.1987.4.129

19. Jhorawat R, Kumari S, Varma S, et al. Preventive role of carvedilol in adriamycin-induced cardiomyopathy. *Indian J Med Res*. 2016;144(5):725. doi:10.4103/ijmr.IJMR_1323_14

20. Speyer JL, Green MD, Kramer E, et al. Protective effect of the bispiperazinedione ICRF-187 against doxorubicin-induced cardiac toxicity in women with advanced breast cancer. *N Engl J Med*. 1988;319(12):745-752. doi:10.1056/NEJM198809223191203

21. Kaya MG, Ozkan M, Gunebakmaz O, et al. Protective effects of nebivolol against anthracycline-induced cardiomyopathy: A randomized control study. *International Journal of Cardiology*. 2013;167(5):2306-2310. doi:10.1016/j.ijcard.2012.06.023

22. Hamidian M, Foroughinia F, Haghighat S, Attar A, Haem E. Protective effects of Panax ginseng against doxorubicin-induced cardiac toxicity in patients with non-metastatic breast cancer: A randomized, double-blind, placebo-controlled clinical trial. *J Oncol Pharm Pract*. 2023;29(6):1306-1316. doi:10.1177/10781552221118530

23. Zhang H, Shen WS, Gao CH, Deng LC, Shen D. Protective effects of salidroside on epirubicin-induced early left ventricular regional systolic dysfunction in patients with breast cancer. *Drugs R D*. 2012;12(2):101-106. doi:10.2165/11632530-000000000-00000

24. Akpek M, Ozdogru I, Sahin O, et al. Protective effects of spironolactone against anthracycline‐induced cardiomyopathy. *European J of Heart Fail*. 2015;17(1):81-89. doi:10.1002/ejhf.196

25. Werida RH, Elshafiey RA, Ghoneim A, Elzawawy S, Mostafa TM. Role of alpha-lipoic acid in counteracting paclitaxel- and doxorubicin-induced toxicities: a randomized controlled trial in breast cancer patients. *Support Care Cancer*. 2022;30(9):7281-7292. doi:10.1007/s00520-022-07124-0

26. Mohamed AL, El-Abd AA, Mohamed HG, Noufal AM, Hennawy BS. Role of Statin Therapy in Prevention of Anthracycline-Induced Cardiotoxicity: A Three Dimentional Echocardiography Study. *Current Problems in Cardiology*. 2024;49(1):102130. doi:10.1016/j.cpcardiol.2023.102130

27. Serageldin MA, Kassem AB, El-Kerm Y, Helmy MW, El-Mas MM, El-Bassiouny NA. The Effect of Metformin on Chemotherapy-Induced Toxicities in Non-diabetic Breast Cancer Patients: A Randomised Controlled Study. *Drug Saf*. 2023;46(6):587-599. doi:10.1007/s40264-023-01305-4

28. Wihandono A, Azhar Y, Abdurahman M, Hidayat S. The Role of Lisinopril and Bisoprolol to Prevent Anthracycline Induced Cardiotoxicity in Locally Advanced Breast Cancer Patients. *Asian Pac J Cancer Prev*. 2021;22(9):2847-2853. doi:10.31557/APJCP.2021.22.9.2847

**Supplement S3: Reference list of studies excluded from quantitative analysis**

| ID | First Author (Year) | title | reason |
| --- | --- | --- | --- |
| 1 | Guthrie, D.1977 | Doxorubicin cardiotoxicity: possible role of digoxin in its prevention | Differences in outcome indicators |
| 2 | Dresdale, A. R.1982 | Prospective randomized study of the role of N-acetyl cysteine in reversing doxorubicin-induced cardiomyopathy | The patient was on other medication |
| 3 | Whittaker, J. A.1984 | Effect of digoxin and vitamin E in preventing cardiac damage caused by doxorubicin in acute myeloid leukemia | Differences in outcome indicators |
| 4 | Mullerleile, U.1986 | Decreased cardiotoxicity of adriamycin by verapamil | review |
| 7 | Blum, R. H.1990 | Modulation of the effect of anthracycline efficacy and toxicity by ICRF-187 | review |
| 8 | Kraft, J.1990 | Effects of Verapamil on Anthracycline-Induced Cardiomyopathy: Preliminary Results of a Prospective Multicenter Trial | Not all studies |
| 9 | Bu'Lock, F. A.1993 | Cardioprotection by ICRF187 against high dose anthracycline toxicity in children with malignant disease | Children |
| 10 | Basser, R. L.1994 | Comparative Study of the Pharmacokinetics and Toxicity of High-Dose Epirubicin With or Without Dexrazoxane in Patients With Advanced Malignancy | Differences in outcome indicators |
| 11 | Iarussi, D.1994 | Protective Effect of Coenzyme Qlo on Anthracyclines Cardiotoxicity: Control Study in Children with Acute Lymphoblastic Leukemia and Non-Hodgkin Lymphoma | Children |
| 13 | Wexler, L. H.1996 | Randomized Trial of the Cardioprotective Agent ICRF-187 in Pediatric Sarcoma Patients Treated With Doxorubicin | Children |
| 14 | Schiavetti, A.1997 | USE OF ICRF-187 FOR PREVENTION OF ANTHRACYCLINE CARDIOTOXICITY I N CHILDREN: Preliminary results | Children |
| 15 | Schuler, D.1997 | SAFETY OF DEXRAZOXANE IN CHILDREN WITH ALL UNDERGOING ANTHRACYCLINE THERAPY: Preliminary Results of a Prospective Pilot Study | Children |
| 17 | Swain, S. M.1997 | Delayed Administration of Dexrazoxane Provides Cardioprotection for Patients With Advanced Breast Cancer Treated With Doxorubicin-Containing Therapy | Not all studies |
| 18 | Fazio, S.1998 | Doxorubicin-Induced Cardiomyopathy Treated with Carvedilol | Case Report |
| 19 | Tallarico, D.2003 | Myocardial Cytoprotection by Trimetazidine Against Anthracycline-Induced Cardiotoxicity in Anticancer Chemotherapy | No randomized grouping |
| 20 | Lipshultz, S. E.2004 | The Effect of Dexrazoxane on Myocardial Injury in Doxorubicin-Treated Children with Acute Lymphoblastic Leukemia | Children |
| 21 | Silber, J. H.2004 | Enalapril to Prevent Cardiac Function Decline in Long-Term Survivors of Pediatric Cancer Exposed to Anthracyclines | Children |
| 22 | Conklin, K. A.2005 | Coenzyme Q10 for Prevention of Anthracycline-Induced Cardiotoxicity | review |
| 23 | Galetta, F.2005 | Effect of epirubicin-based chemotherapy and dexrazoxane supplementation on QT dispersion in non-Hodgkin lymphoma patients | Differences in outcome indicators |
| **24** | **Nakamae, H.2005** | Notable Effects of Angiotensin II Receptor Blocker, Valsartan, on Acute Cardiotoxic Changes after Standard Chemotherapy with Cyclophosphamide, Doxorubicin, Vincristine, and Prednisolone | Incomplete data |
| 25 | Paiva, M. G.2005 | Cardioprotective Effect of Dexrazoxane During Treatment With Doxorubicin: A Study Using Low-Dose Dobutamine Stress Echocardiography{ | Children |
| 27 | Kalay, N.2006 | Protective Effects of Carvedilol Against Anthracycline-Induced Cardiomyopathy | No indication of the types of cancers included |
| 29 | Cadeddu, C.2010 | Protective effects of the angiotensin II receptor blocker telmisartan on epirubicin-induced inflammation, oxidative stress, and early ventricular impairment | Not all studies |
| 30 | Choi, H. S.2010 | Dexrazoxane for Preventing Anthracycline Cardiotoxicity in Children with Solid Tumors | Children |
| 32 | Lipshultz, S. E.2010 | Assessment of dexrazoxane as a cardioprotectant in doxorubicin-treated children with high-risk acute lymphoblastic leukaemia: long-term follow-up of a prospective, randomised, multicentre trial | Children |
| 34 | Salehi, R.2011 | Protective Effect of Carvedilol in Cardiomyopathy Caused by Anthracyclines in Patients Suffering from Breast Cancer and Lymphoma | Children |
| 35 | El-Shitany, N. A.2012 | Protective Effect of Carvedilol on Adriamycin-Induced Left Ventricular Dysfunction in Children With Acute Lymphoblastic Leukemia | Children |
| 37 | Akpek, M.2013 | Sipironolactone prevents the heart againts anthracycline cardiotoxicity | Review |
| 38 | Bosch, X.2013 | Enalapril and Carvedilol for Preventing Chemotherapy-Induced Left Ventricular Systolic Dysfunction in Patients With Malignant Hemopathies | Blood disease |
| 41 | Radulescu, D.2013 | Can the epirubicin cardiotoxicity in cancer patients be prevented by angiotensin converting enzyme inhibitors? | No randomized grouping |
| 42 | Broeyer, F. J.2014 | Evaluation of Lecithinized Human Recombinant Super Oxide Dismutase as Cardioprotectant in Anthracycline-treated Breast Cancer Patients 1 | Incomplete data |
| 44 | Negishi, K.2014 | Use of speckle strain to assess left ventricular responses to cardiotoxic chemotherapy and cardioprotection | No cardiac protection |
| 46 | Chotenimitkhun, R.2015 | Chronic Statin Administration May Attenuate Early Anthracycline-Associated Declines in Left Ventricular Ejection Function | The patient was on other medication |
| 47 | Sun, F.2015 | Dexrazoxane Protects Breast Cancer Patients With Diabetes From Chemotherapy-Induced Cardiotoxicity | Diabetes |
| 48 | Witteles, R. M.2015 | Myocardial Protection During Cardiotoxic Chemotherapy | Review |
| 49 | Zhang, H. Y.2015 | Coenzyme Complex Decreased Cardiotoxicity When Combined with Chemotherapy in Treating Elderly Patients with Gastrointestinal Cancer | Non-anthracycline-based chemotherapy |
| 50 | Armenian, S. H.2016 | Rationale and design of the Children’s Oncology Group (COG) study ALTE1621: a randomized, placebo-controlled trial to determine if low-dose carvedilol can prevent anthracycline-related left ventricular remodeling in childhood cancer survivors at high risk for developing heart failure | Children |
| 51 | Asselin, B. L.2016 | Cardioprotection and Safety of Dexrazoxane in Patients Treated for Newly Diagnosed T-Cell Acute Lymphoblastic Leukemia or Advanced-Stage Lymphoblastic Non-Hodgkin Lymphoma: A Report of the Children’s Oncology Group Randomized Trial Pediatric Oncology Group 9404 | Children |
| 53 | Chow, E. J.2016 | Effect of Dexrazoxane on Heart Function Among LongTerm Survivors of Childhood Leukemia and Lymphoma: A Report from the Children's Oncology Group (COG) | Cohort study |
| 54 | Gulati, G.2016 | Prevention of cardiac dysfunction during adjuvant breast cancer therapy (PRADA): a 2 3 2factorial, randomized, placebo-controlled, double-blind clinical trial of candesartan and metoprolol | A factorial clinical trial |
| 56 | Druk, I.2017 | Druk-20Prevention with simvastatin of anthracycline-induced cardiotoxicity during chemotherapy for breast cancer17-Prevention with simvastatin of anthr.pdf | Summary only |
| 57 | Guglin, M.2017 | Lisinopril or Coreg CR in reducing cardiotoxicity in women with breast cancer receiving trastuzumab: A rationale and design of a randomized clinical trial | Non-anthracycline-based chemotherapy |
| 58 | Gulati, G.2017 | Neurohormonal Blockade and Circulating Cardiovascular Biomarkers During Anthracycline Therapy in Breast Cancer Patients: Results From the PRADA (Prevention of Cardiac Dysfunction During Adjuvant Breast Cancer Therapy) Study | Not all studies |
| 60 | Abuosa, A. M.2018 | Prophylactic use of carvedilol to prevent ventricular dysfunction in patients with cancer treated with doxorubicin | No indication of the types of cancers included |
| 61 | Aggarwal, S.2018 | Long-term cardioprotective effects of dexrazoxane infusion during anthracycline chemotherapy: a children's oncology group speckle echocardiography study | Children |
| 62 | Askolskyi, A.2018 | Predicting the occurrence and prevention of early anthracycline cardiotoxicity of chemotherapy in patients with breast cancer | Observational studies |
| 64 | Cardinale, D.2018 | Anthracycline-induced cardiotoxicity: A multicenter randomised trial comparing two strategies for guiding prevention with enalapril: The International CardioOncology Society-one trial | No indication of the types of cancers included |
| 66 | Fadol, A. P.2018 | Management of chemotherapyinduced Left ventricular Dysfunction and Heart Failure in Patients With cancer While Undergoing cancer treatment: the MD Anderson Practice | A descriptive study |
| 67 | Gupta, V.2018 | Role of ACE inhibitors in anthracycline-induced cardiotoxicity: A randomized, double-blind, placebo-controlled trial | Children |
| 68 | Heck, S. L.2018 | Effect of candesartan and metoprolol on myocardial tissue composition during anthracycline treatment: the PRADA trial | The patient was on other medication |
| 69 | Davis, M. K.2019 | Effect of Eplerenone on Diastolic Function in Women Receiving Anthracycline-Based Chemotherapy for Breast Cancer | Differences in outcome indicators |
| 70 | de la Rosa Oliva, F.2019 | Effects of omega-3 fatty acids supplementation on neoadjuvant chemotherapyinduced toxicity in patients with locally advanced breast cancer: a randomized, controlled, double-blinded clinical trial | Differences in outcome indicators |
| 71 | Georgakopoulos, P.2019 | The Role of Metoprolol and Enalapril in the Prevention of Doxorubicin-induced Cardiotoxicity in Lymphoma Patients | Not all studies |
| 72 | Koonarat, A.2019 | PB1795 CARDIOPROTECTIVE EFFECTS OF ATORVASTATIN IN B-CELL LYMPHOMA PATIENTS RECEIVING RCHOP REGIMEN, A RANDOMIZED CONTROLLED TRIAL..pdf | Summary only |
| 73 | Kopp, L. M.2019 | Effects of dexrazoxane on doxorubicinrelated cardiotoxicity and second malignant neoplasms in children with osteosarcoma: a report from the Children’s Oncology Group | Children |
| 74 | Minotti, G.2019 | Pharmacology of Ranolazine versus Common Cardiovascular Drugs in Patients with Early Diastolic Dysfunction Induced by Anthracyclines or Nonanthracycline Chemotherapeutics: A Phase 2b Minitrial | Non-anthracycline-based chemotherapy |
| 75 | Scott, J. M. | Effects of adjunct testosterone on cardiac morphology and function in advanced cancers: an ancillary analysis of a randomized controlled trial | Non-anthracycline-based chemotherapy |
| 76 | Ayub-Ferreira, S. M.2020 | CARVEDILOL FOR PREVENTION OF CHEMOTHERAPY-INDUCED CARDIOTOXICITY: FINAL RESULTS OF THE PROSPECTIVE, RANDOMIZED, DOUBLE-BLIND, PLACEBO CONTROLLED CECCY TRIAL | A factorial clinical trial |
| 77 | Barletta, G.2020 | Anthracycline and trastuzumab-induced subclinical cardiac damage and its prevention in the SAFE trial. Myocardial strain imaging and 3D echo interim analysis data | Summary only |
| 78 | Bolli, R.2020 | A Phase I Study of Allogeneic Mesenchymal Stem Cell Therapy in Patients with Heart Failure Secondary to Anthracycline-induced Cardiomyopathy: the Cctrn Stem Cell Injection in Cancer Survivors (Seneca) Trial | Summary only |
| 79 | Brown, S. A.2020 | The Role of Angiotensin-Converting Enzyme Inhibitors and b-Blockers in Primary Prevention of Cardiac Dysfunction in Breast Cancer Patients | Review |
| 80 | Filomena, D.2020 | Echocardiographic long-term follow-up of adult survivors of pediatric cancer treated with Dexrazoxane-Anthracyclines association | Children |
| 81 | Gregorietti, V.2020 | Use of Sacubitril/valsartan in patients with cardio toxicity and heart failure due to chemotherapy | The patient was on other medication |
| 82 | Hagag, A. A.2020 | Protective role of black seed oil in doxorubicin-induced cardiac toxicity in children with acute lymphoblastic leukemia | Children |
| 84 | Martha, J. W.2020 | The effect of prophylactic carvedilol on subclinical left ventricular dysfunction after 1 cycle FAC chemotherapy in breast cancer patients | Differences in outcome indicators |
| 85 | Słowik, A.2020 | Anthracycline-induced cardiotoxicity prevention with angiotensin-converting enzyme inhibitor ramipril in women with low-risk breast cancer: results of a prospective randomized study | The patient was on other medication |
| 86 | Zalat, Z.2020 | Evaluation of the cardioprotective effects of l-carnitine and silymarin in cancer patients receiving anthracycline-containing chemotherapy | Summary only |
| 87 | Heck, S. L.2021 | Prevention of Cardiac Dysfunction During Adjuvant Breast Cancer Therapy (PRADA) | A factorial clinical trial |
| 89 | Rizka, A.2021 | Role of angiotensin-converting enzyme inhibitors on changes in troponin levels in breast cancer with anthracycline chemotherapy | The patient was on other medication |
| 91 | Zito, C.2021 | Anthracyclines and regional myocardial damage in breast cancer patients. A multicentre study from the Working Group on Drug Cardiotoxicity and Cardioprotection, Italian Society of Cardiology (SIC) | No cardiac protection |
| 92 | Attar, A.2022 | Sildenafil for Primary Prevention of Anthracycline-Induced Cardiac Toxicity: A Phase I/II Randomized Clinical Trial, SILDAT-TAHA6 Trial | No indication of the types of cancers included |
| 88 | Lee, M.2021 | Candesartan and carvedilol for primary prevention of subclinical cardiotoxicity in breast cancer patients without a cardiovascular risk treated with doxorubicin | Not all studies |
| 94 | de Barros Wanderley, M. R.2022 | Plasma biomarkers reflecting high oxidative stress in the prediction of myocardial injury due to anthracycline chemotherapy and the effect of carvedilol: insights from the CECCY Trial | Not all studies |
| 95 | El-Bassiouny, N. A.2022 | The Cardioprotective Effect of Vitamin D in Breast Cancer Patients Receiving Adjuvant Doxorubicin Based Chemotherapy | Differences in outcome indicators |
| 94 | El Amrousy, D. | Omega 3 fatty acids can reduce early doxorubicin-induced cardiotoxicity in children with acute lymphoblastic leukemia | Children |
| 97 | Maier, R. H.2022 | Preventing cardiotoxicity in patients with breast cancer and lymphoma: protocol for a multicentre randomised controlled trial (PROACT) | Program |
| 98 | Sławiński, G.2022 | Effective cardioprotection with early initiation of sacubitrilvalsartan in a patient with breast cancer and cancer treatment-induced heart failure | Case report |
| 100 | Chow, E. J.2023 | Dexrazoxane and Long-Term Heart Function in Survivors of Childhood Cancer | Children |
| 104 | Karvandi, M.2023 | Evaluation of the Effect of Carvedilol in Preventing Right Ventricular Dysfunction in Breast Cancer Patients Receiving Anthracycline | Differences in outcome indicators |
| 105 | Li, B.2023 | PEG-conjugated bovine haemoglobin enhances efficiency of chemotherapeutic agent doxorubicin with alleviating DOX-induced splenocardiac toxicity in the breast cancer | Review |
| 106 | Neilan, T. G.2023 | Atorvastatin for Anthracycline-Associated Cardiac Dysfunction: The STOP-CA Randomized Clinical Trial | The patient was on other medication |
| 107 | Osataphan, N.2023 | Effects of metformin and donepezil on the prevention of doxorubicin-induced cardiotoxicity in breast cancer: a randomized controlled trial | The patient was on other medication |
| 108 | Rahimi, K.2023 | Cardioprotective effects of deferoxamine in acute and subacute cardiotoxicities of doxorubicin: a randomized clinical trial | Children |
| 110 | Thavendiranathan, P.2023 | Statins to prevent early cardiac dysfunction in cancer patients at increased cardiotoxicity risk receiving anthracyclines | Patients with combined cardiac risk factors |
| 111 | Armenian, S. H.2023 | Effect of carvedilol versus placebo on cardiac function in anthracycline-exposed survivors of childhood cancer (PREVENT-HF): a randomised, controlled, phase 2b trial | Children |
| 112 | Chen, C. Y.2023 | Statin Use Is Associated With Reduced Heart Failure and Risk of Death in Non-Hodgkin Lymphoma | Cohort study |

**Supplement S4: Characteristics of studies and patients’ baseline**

|  | Author, year | Study location | Design | Code RCT Or Study | Tumor | Size, n (I/C) | Male/female | Mean age (years) (I/C) | Outcome |
| --- | --- | --- | --- | --- | --- | --- | --- | --- | --- |
| 1 | Speyer, J. L. 1988 | America | Prospective randomized clinical trial | —— | Breast cancer | 92（47/45） | 0/92 | 55/52 | Cardiac Events |
| 2 | Venturini, M.1996 | Italy | Randomized multiinstitutional controlled clinical study | —— | Metastatic, Locally advanced (III B) or Inflammatory breast cancer | 160（82/78) | —— | 54/55 | Cardiac Events |
| 3 | Marty, M.2006 | Czech Republic, France, Germany, Poland, South Africa and Spain | Multicenter, international, open-label, randomized, controlled phase III study | —— | Breast cancer | 164（85/79） | 0/164 | 52.3/51 | Cardiac Events |
| 4 | Georgakopoulos, P.2010 | Greece | A prospective, parallelgroup, randomized, controlled study with 36-month follow-up | —— | Hodgkin lymphoma (HL) and Non-Hodgkin lymphoma (NHL) | （42/40） | 43/39 | 51.0/49.1 | LVEF; Cardiac Events; LVESD; LVEDD; E/A |
|  |  |  |  |  |  | （43/40） | 43/40 | 47.4/49.1 |  |
| 5 | Jo, S. H.2013 | Korea | Prospective randomized open label controlled trial | —— | Breast cancer、Lymphoma | 103（50/53） | 5/98 | 51.5/48.3 | LVEF; Cardiac Events; LVESD; LVEDD; E/A |
| 6 | Jhorawat, R.2016 | India | —— | —— | Lymphoreticular malignancy | 54（27/27） | 41/13 | 43.9/38.7 | LVEF; E/A |
| 7 | Cochera, F.2018 | Romania | Prospective study | —— | Breast cancer | 60（30/30） | 0/60 | 53/52 | LVEF; LVESD; LVEDD; E/A |
| 8 | Wihandono, A.2021 | Indonesia | open-label prospective randomized controlled trial | —— | Breast cancer | 51（26/25） | 0/51 | 44.5/50.8 | LVEF |
| 9 | Li, X.2022 | China | Randomized controlled trial | —— | Breast cancer, Malignant lymphoma | 223（113/110） | 99/124 | 57/58 | LVEF |
| 10 | Ciburiene, E.2023 | Lithuania | Prospective, Randomized, Open-Label Clinical Trial | NCT04030546/2019-000661-20 | Breast cancer, Sarcoma | 48（21/27） | 0/48 | 47.8/48 | LVEF |
| 11 | Henriksen, P. A.2023 | United Kingdom | Multicenter, prospective, randomized, open-label, blinded end-point trial, | —— | Breast cancer, Non-Hodgkin lymphoma | 57（29/28） | 12/45 | 54/54 | LVEF; Cardiac Events |
| 12 | Serageldin, M. A.2023 | Egypt | Randomised Controlled Study | NCT04170465 | Breast cancer | 70（35/35） | 0/70 | —— | LVEF |
| 13 | Moustafa, I.2024 | Saudi Arabia | Prospective, randomized controlled study | —— | Breast cancer | 74（35/39） | 0/74 | 52.51/51.46 | Cardiac Events |
| 14 | Milei, J.1987 | Argentina | Pilot, double-blind trial | —— | Breast carcinoma, Lung carcinoma, Ovarian carcinom etc. | 26 (13/13) | 9/17 | 63.8/59.7 | Cardiac Events |
| 15 | Swain, S. M.1997 | America | Multicenter, double-blind studies | —— | Breast cancer | 534（249/285) | 0/534 | 56/54 | Cardiac Events |
| 16 | Mantovani, G.2011 | Italy | One-institution ‘independent’, randomized, PLA-controlled trial | —— | Endometrium、Salivary gland、Non-Hodgkin lymphoma、Breast、Ovary、Lung (NSCLC) | 49（25/24） | 12/37 | LVEF | LVEF; E/A |
| 17 | Zhang, H.2012 | China | —— | —— | Breast cancer | 60（30/30） | 0/60 | 51/52 | LVEF |
| 18 | Kaya, M. G.2013 | Turkey | Randomized, double-blind, placebo-controlled clinical study | —— | Breast cancer | 45（27/18） | 0/45 | 51.4/50.5 | LVEF; LVESD; LVEDD; E/A |
| 19 | Elitok, A.2014 | Turkey | Prospective and randomized study | —— | Breast cancer | 80（40/40） | 0/80 | 54.3/52.9 | LVEF; LVESD; LVEDD; E/A |
| 20 | Akpek, M.2015 | Turkey | Prospective, randomized, placebo-controlled, and double-blind study | NCT02053974 | Breast cancer | 83（43/40） | 0/83 | 50.0/50.6 | LVEF; LVESD; LVEDD |
| 21 | Beheshti, A. T.2016 | Iran | Double-blind pilot randomized control trial | IRCT2013093012924N1 | Breast cancer | 70（30/40） | 0/70 | 42.0/39.9 | LVEF |
| 22 | Janbabai, G.2017 | Iran | Randomized, single-blind, and placebo-controlled trial | —— | Breast、Wilms tumor、Lung cancer、Bone sarcoma、Hodgkin’s lymphoma、 | 69（34/35） | 5/64 | 47.76/47.06 | E/A |
| 23 | Avila, M. S.2018 | Brazil | Prospective, double-blind, randomized, placebo-controlled study | NCT01724450 | Breast cancer | 192（96/96） | 0/192 | 50.8/52.9 | LVEF; Cardiac Events; LVESD; LVEDD |
| 24 | Hao, W.2020 | China | Randomized, double-blind, placebo-controlled trial | ChiCTR-IPR-16009256 | Breast cancer | 125（61/64） | 0/125 | 50/49 | Cardiac Events |
| 25 | Cao, S.2022 | China | Randomized placebo-based trial | ChiCTR1900022394 | Breast cancer | 50（25/25） | 0/50 | 48.5/47.5 | LVEF |
| 26 | Werida, R. H.2022 | Egypt | Randomized double-blind placebo-controlled prospective study | NCT03908528 | Breast cancer | 64（32/32） | 0/64 | 48.19/49.56 | LVEF |
| 27 | Hamidian, M.2023 | Iran | Randomized, double-blind, placebo-controlled clinical trial | IRCT20141227020441N7 | Breast cancer | 30（15/15） | 0/30 | 44.0/43.3 | LVEF; Cardiac Events; LVESD; LVEDD |
| 28 | Mohamed, A. L.2023 | Egypt | Prospective, randomized, single-blind, placebo-controlled trial | —— | Breast cancer | 100（50/50） | 0/100 | 47.84/49.8 | LVEF; LVESD |

**Supplement S5: Main characteristics of included trials**

| **Record number** | **Author, year** | **Treatment** | **Route of administration** | **Administration time** | **Dosage of administration** | **Intervention** | **Control** |
| --- | --- | --- | --- | --- | --- | --- | --- |
| 1 | Speyer, J. L. 1988 | ICRF-187 | Intravenous injection | 30 minutes before FDC | 1000 mg/m2 | Chemotherapy＋ICRF-187 | Chemotherapy |
| 2 | Venturini, M.1996 | Dexrazoxane | Intravenous injection | 30 minutes before epirubicin. | 600 mg/m2 | Chemotherapy＋Dexrazoxane | Chemotherapy |
| 3 | Marty, M.2006 | Dexrazoxane | Intravenous injection | 30 minutes before anthracycline | Dexrazoxane was infused at a 20:1 dexrazoxane:doxorubicin dose ratio, or at a 10:1 dexrazoxane: epirubicin dose ratio | Chemotherapy＋Dexrazoxane | Chemotherapy |
| 4 | Georgakopoulos, P.2010 | Metoprolol | Take orally | During chemotherapy | 88.8mg/d(Average cumulative dose） | Chemotherapy＋Metoprolol | Chemotherapy |
|  |  | Enalapril | Take orally | During chemotherapy | 11mg/d(Average cumulative dose） | Chemotherapy＋Enalapril | Chemotherapy |
| 5 | Jo, S. H.2013 | N-Acetylcysteine | Take orally | Every 8 hours starting before and ending after the intravenous infusion of anthracycline in all chemotherapy cycles (3-6) | 1200 mg/trip | Chemotherapy＋NAC | Chemotherapy |
| 6 | Jhorawat, R.2016 | Carvedilol | Take orally | Started before chemotherapy and maintained for 6 months while on chemotherapy | 12.5mg/day | Chemotherapy＋Carvedilol | Chemotherapy |
| 7 | Cochera, F.2018 | Nebivolol | —— | During chemotherapy | 5 mg/day | Chemotherapy＋nebivolol | Chemotherapy |
| 8 | Wihandono, A.2021 | Lisinopril and Bisoprolol | Take orally | Started simultaneously 24 h before the first cycle of chemotherapy | The average dose of lisinopril was 7.92 mg/day while the bisoprolol dose was 6.47 ± 1.07 mg/ day. | Chemotherapy＋Lisinopril and Bisoprolol | Chemotherapy |
| 9 | Li, X.2022 | Astragalus polysaccharide | Intravenous injection | 2 weeks as a course of treatment, 2 courses of treatment | 500 mg astragalus polysaccharides injection was added into 500 mL normal saline,once a day, | Chemotherapy＋astragalus polysaccharide | Chemotherapy |
| 10 | Ciburiene, E.2023 | Ivabradine | —— | During chemotherapy | 5 mg twice a day | Chemotherapy＋Ivabradine | Chemotherapy |
| 11 | Henriksen, P. A.2023 | Combined treatment with carvedilol and candesartan | —— | Allocated within 14 days of randomisation and continued until completion or withdrawal from the study | Candesartan, starting dose 8 mg once daily, increasing to 16 and 32 mg, Carvedilol, starting dose 6.25 mg twice daily, increasing to 12.5 and 25 mg twice daily | Chemotherapy＋carvedilol and candesartan | Chemotherapy |
| 12 | Serageldin, M. A.2023 | Metformin | Take orally | During chemotherapy | 1700 mg/day | Chemotherapy＋Metformin | Chemotherapy |
| 13 | Moustafa, I.2024 | Vitamin E and levocarnitine | Vitamin E orally, levocanidin orally and intravenously | Intravenous leucovorin was administered in the hospital prior to chemotherapy. Patient self-administered doses of vitamin E and oral leucovorin (days 2-21) | Vitamin E (600 mg three times daily) and leucovorin (day 1 = 3000 mg IV; days 2-21 = 300 mg four times daily) | Chemotherapy＋EL | Chemotherapy |
| 14 | Milei, J.1987 | Prenylamine | Take orally | During chemotherapy | 200mg/ day (2 capsules/day) | Chemotherapy＋Prenylamine | Chemotherapy＋placebo |
| 15 | Swain, S. M.1997 | Dexrazoxane | Slow IV push or rapid-drip IV infusion | Drug was administered between 15 and 30 minutes before doxorublcin | 50 mL/m2 | Chemotherapy＋Dexrazoxane | Chemotherapy＋placebo |
| 16 | Mantovani, G.2011 | Telmisartan | Take orally | 1 week before the beginning of EPI treatment and up to 6 months after EPI discontinuation | 40 mg/d | Chemotherapy＋Telmisartan | Chemotherapy＋placebo |
| 17 | Zhang, H.2012 | Salidroside | —— | Beginning the therapy 1 week before the start of chemotherapy and continuing for the entire period of epirubicin administration | 600mg/day | Chemotherapy＋Salidroside | Chemotherapy＋placebo |
| 18 | Kaya, M. G.2013 | Nebivolol | Take orally | The morning before chemo for seven days | 5 mg/day | Chemotherapy＋nebivolol | Chemotherapy＋placebo |
| 19 | Elitok, A.2014 | Carvedilol | Take orally | 12.5 mg before computed tomography followed by 12.5 mg maintenance dose during chemotherapy for 6 months. | —— | Chemotherapy＋Carvedilol | Chemotherapy＋placebo |
| 20 | Akpek, M.2015 | Spironolactone | —— | Treatment started 1 week before the start of chemotherapy and stopped three weeks after the end of the chemotherapy regimen | 25mg/day | Chemotherapy＋spironolactone | Chemotherapy＋placebo |
| 21 | Beheshti, A. T.2016 | Carvedilol | Take orally | Starting 10 days before chemotherapy and ending on the 10th day after the end of chemotherapy | 6.25 mg twice daily | Chemotherapy＋Carvedilol | Chemotherapy＋placebo |
| 22 | Janbabai, G.2017 | Enalapril | —— | Started at least 24 hours before the first cycle of chemotherapy and maintained for the duration of chemotherapy for 6 months | 5 mg twice daily | Chemotherapy＋Enalapril | Chemotherapy＋placebo |
| 23 | Avila, M. S.2018 | Carvedilol | —— | On the first day of chemotherapy | Maximum dose of 25 mg every 12 hours | Chemotherapy＋Carvedilol | Chemotherapy＋placebo |
| 24 | Hao, W.2020 | Platycodon grandiflorum | Take orally | Maintained for 6 cycles during chemotherapy. | 6mg/day | Chemotherapy＋Platycodon grandiflorum | Chemotherapy＋placebo |
| 25 | Cao, S.2022 | Hong Huang Decoction | Take orally | During chemotherapy | Each patient ingested 200 mL HDD as a split dose, | Chemotherapy＋Hong Huang Decoction | Chemotherapy＋placebo |
| 26 | Werida, R. H.2022 | Alpha-lipoic acid | Take orally | During chemotherapy | 600 mg/day | Chemotherapy＋alpha-lipoic acid | Chemotherapy＋placebo |
| 27 | Hamidian, M.2023 | Panax ginseng | Take orally | Administered on the same day as the first DOX administration and continued for one week after completion of DOX treatment | 1 g/day | Chemotherapy＋Panax ginseng | Chemotherapy＋placebo |
| 28 | Mohamed, A. L.2023 | Atorvastatin | Take orally | During chemotherapy | 40 mg/d | Chemotherapy＋Atorvastatin | Chemotherapy＋placebo |

**Supplement S6: Risk of bias assessment of the eligible randomized controlled trials**

**
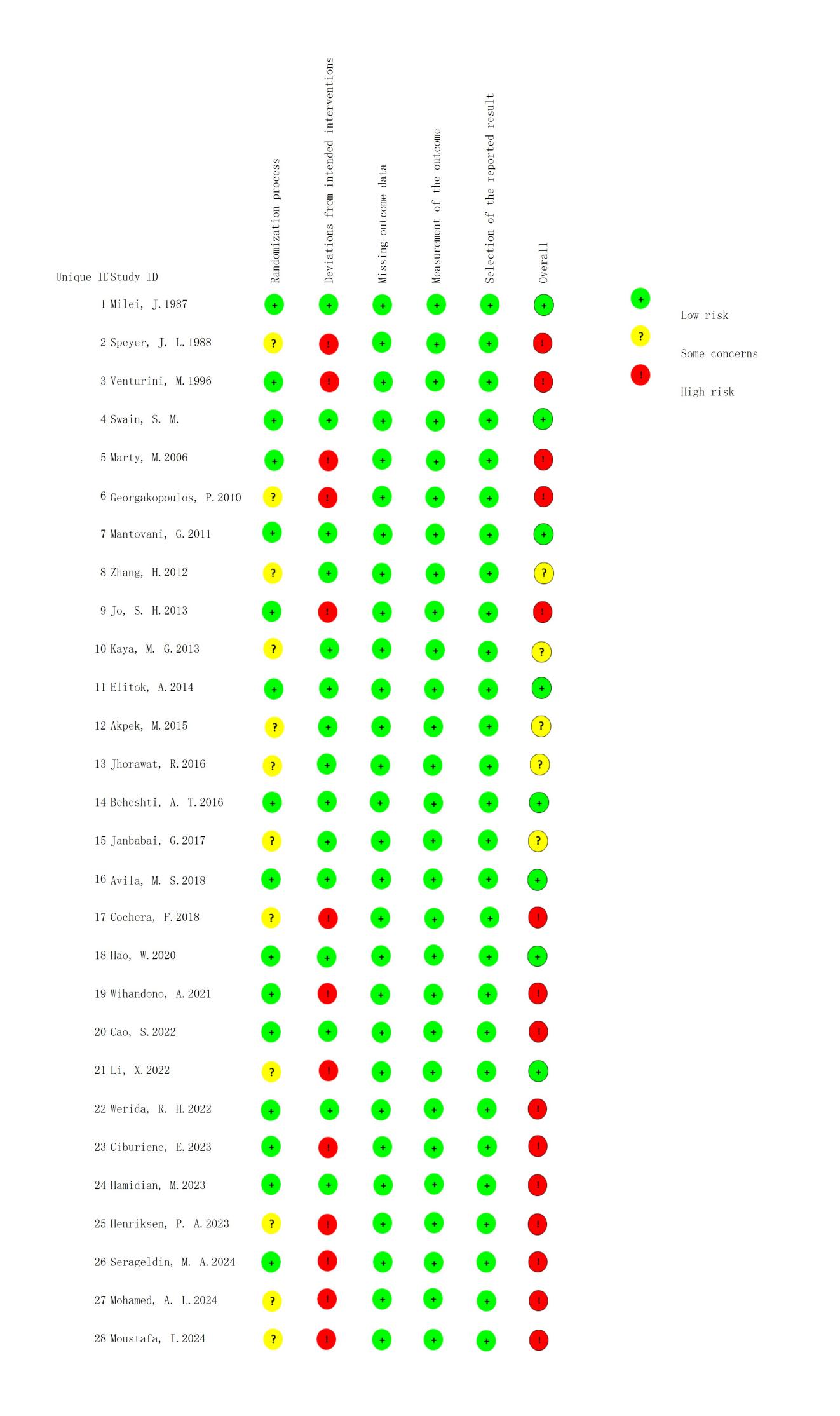
**

**
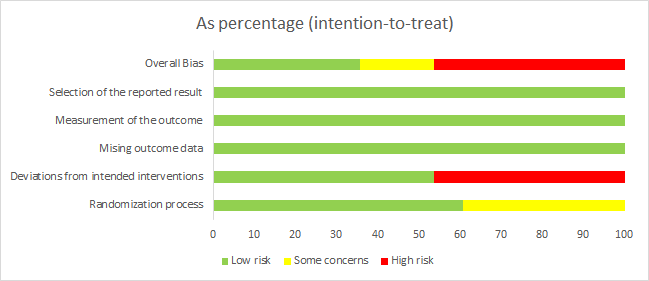
**

**Supplement S7: Meta-regression results**

**Left ventricular ejection fraction (LVEF)**

**
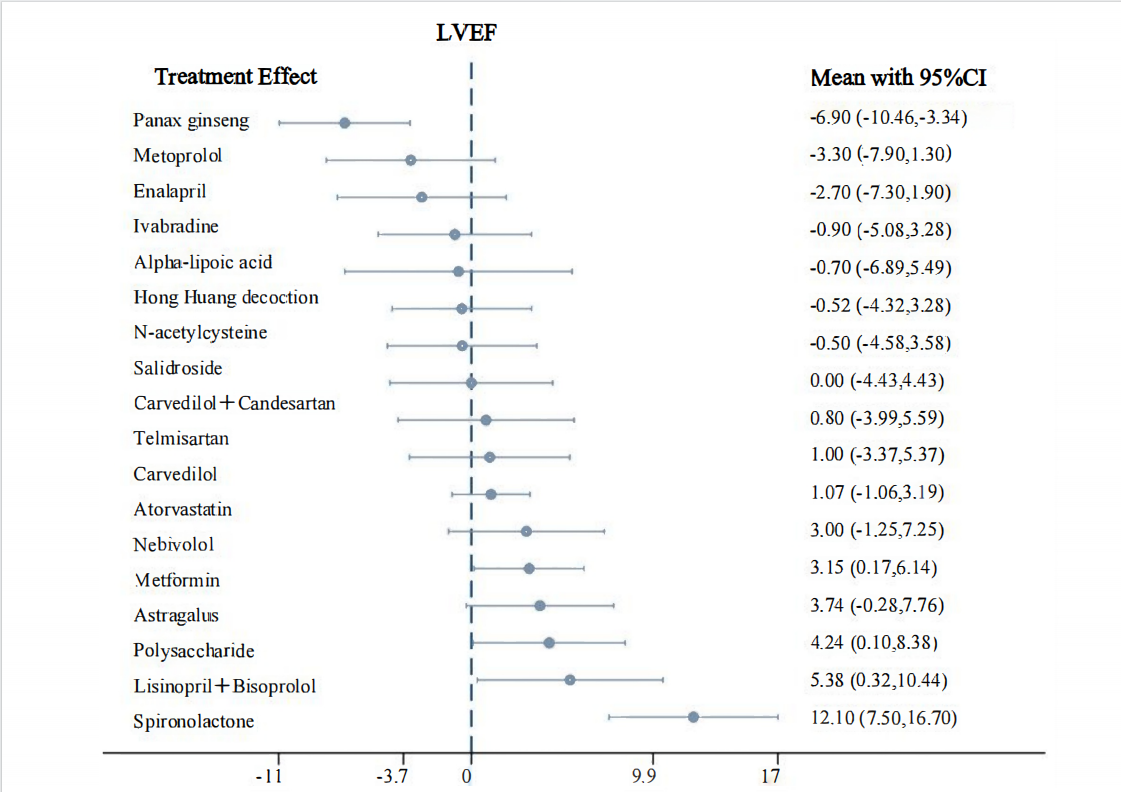
**

**Cardiac Events(A 0.5 correction has been made for zero events)**

**
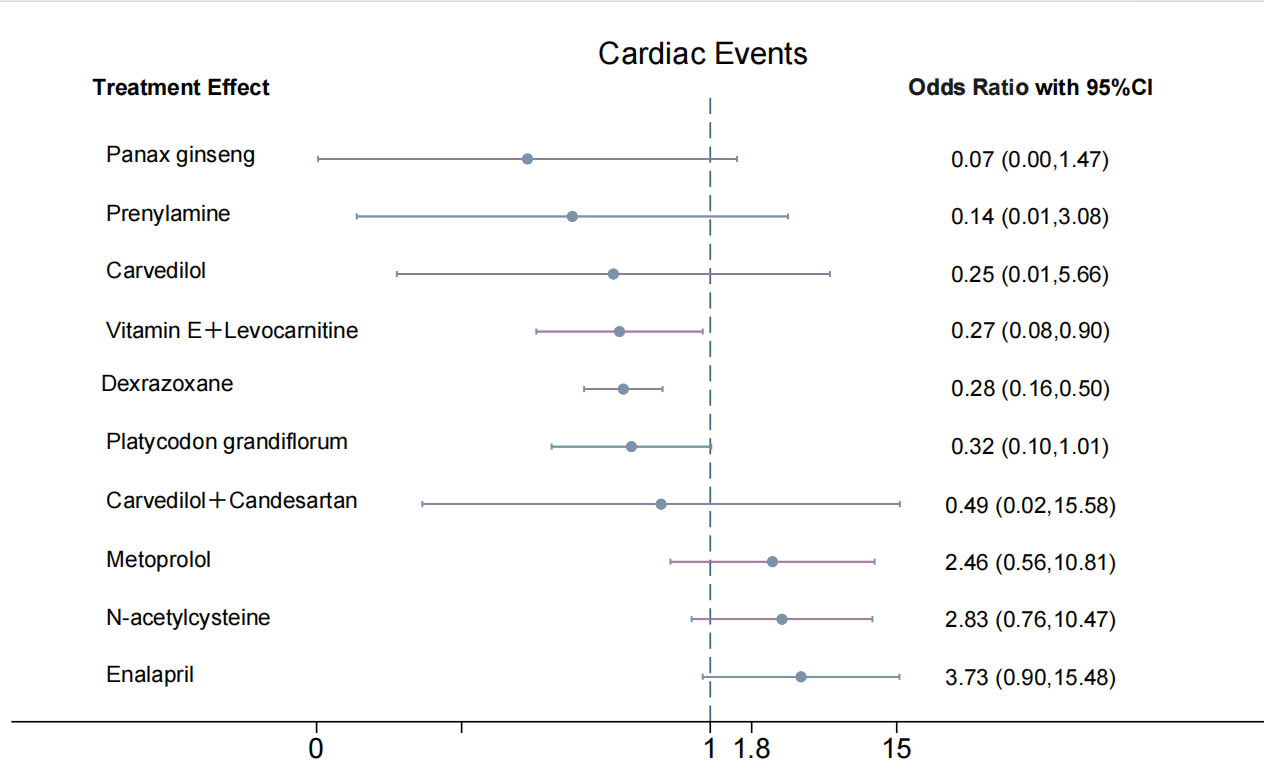
**

**The ratio of mitral annular diastolic peak velocity to atrial systolic velocity (E/A)**

**
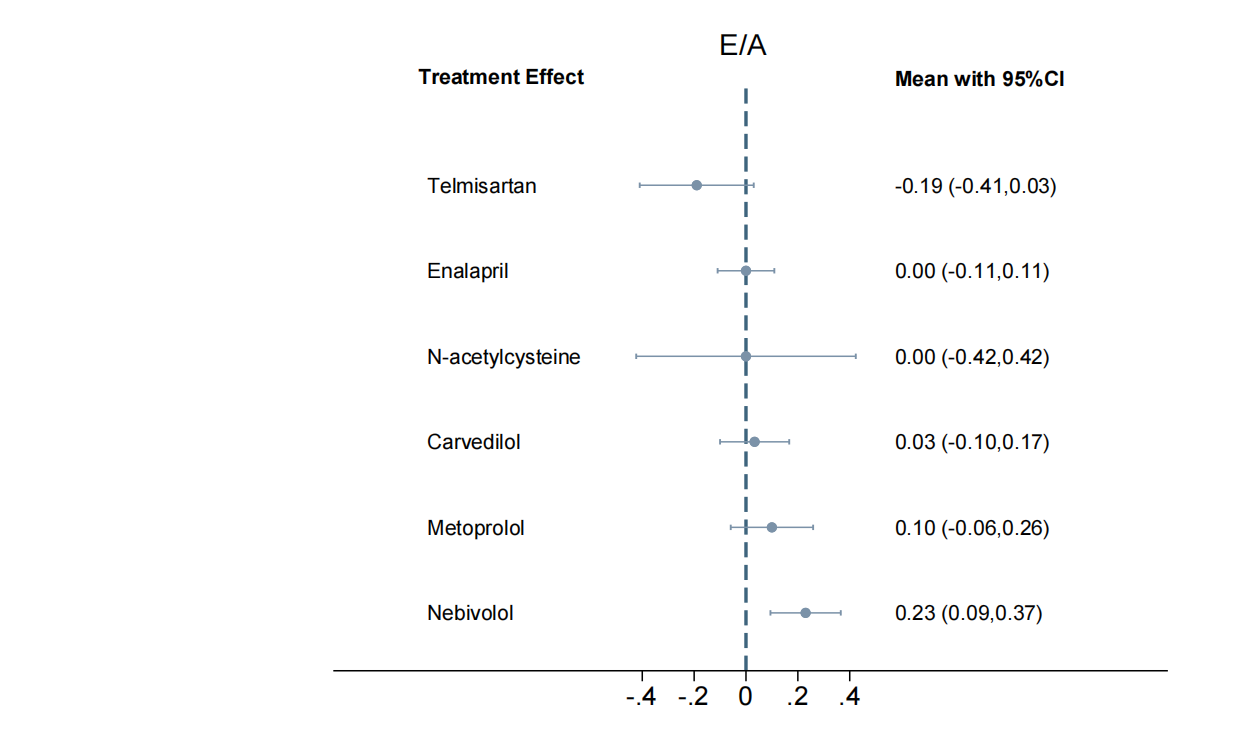
**

**Left ventricular end-diastolic diameter (LVEDD)**

**
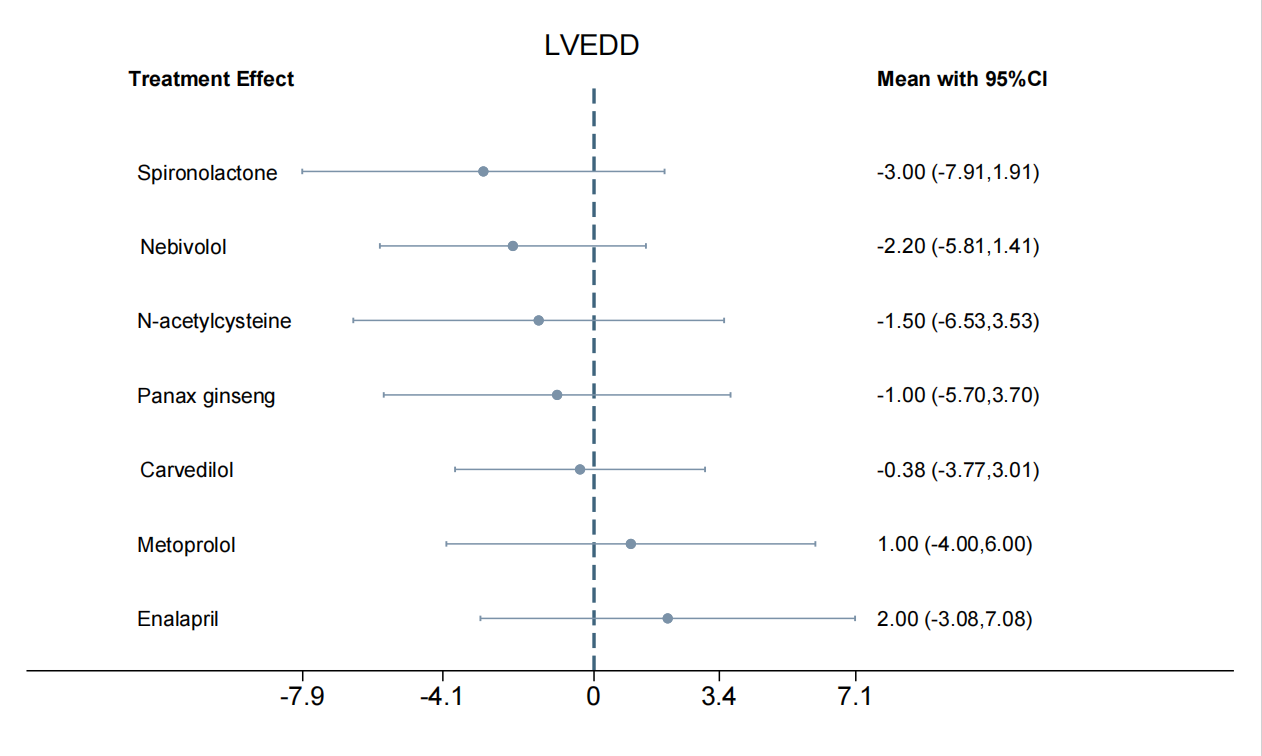
**

**Left ventricular end-systolic diameter (LVESD)**

**
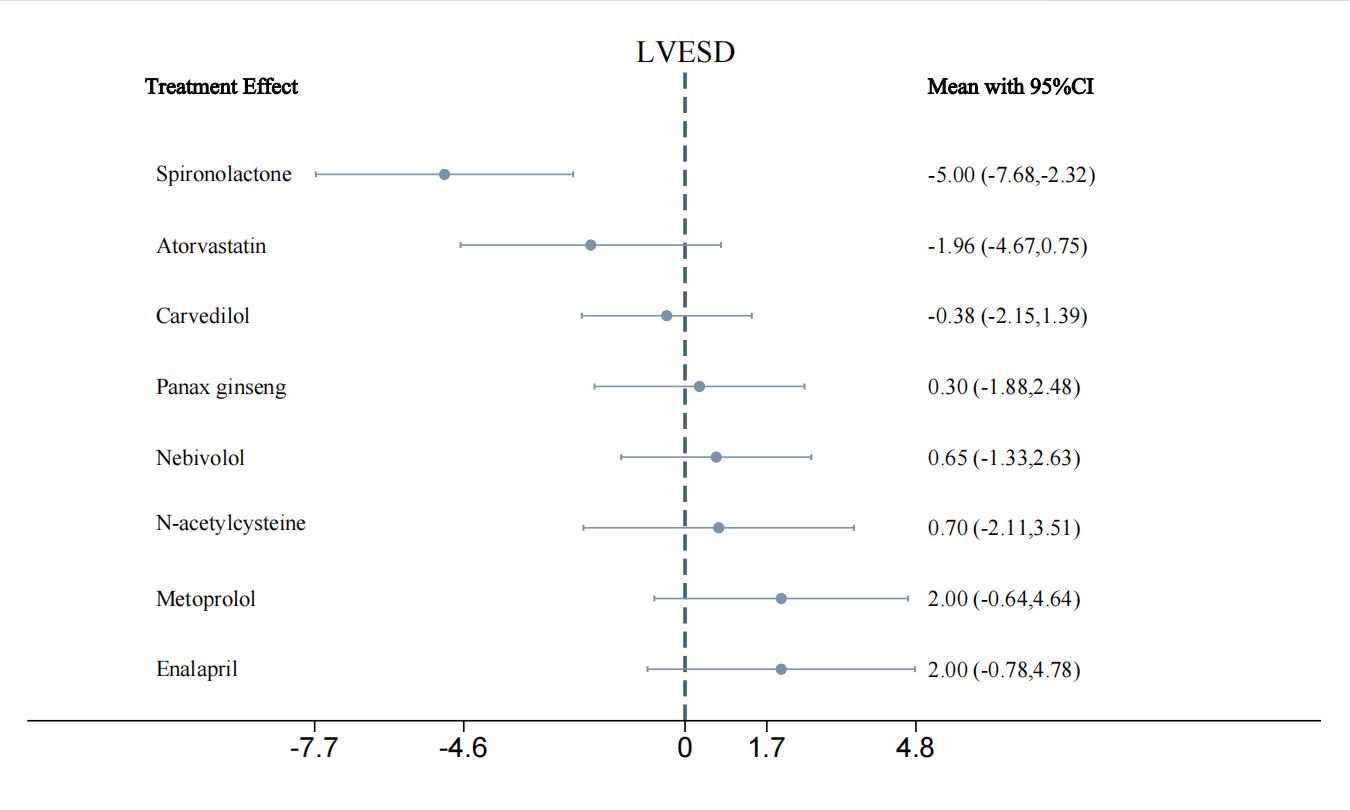
**

**Supplement S8: The surface under the cumulative ranking (SUCRA)**

**Left ventricular ejection fraction (LVEF)**

**
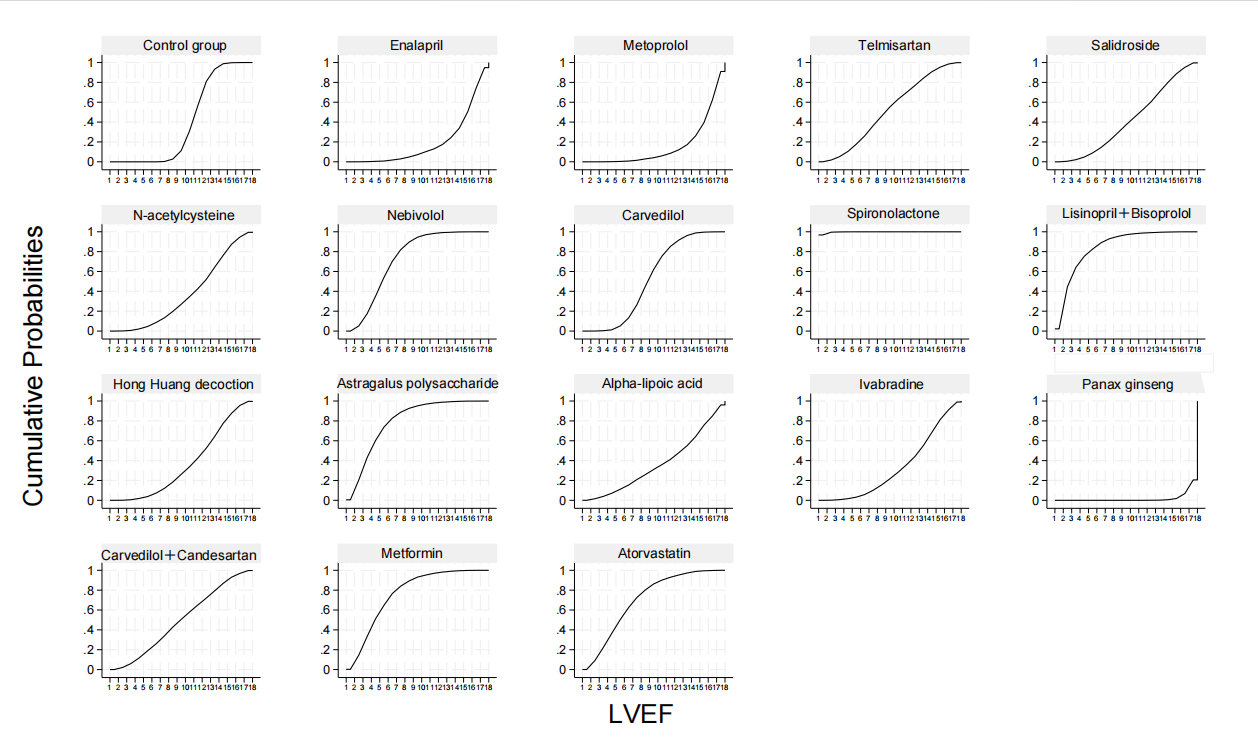
**

**Cardiac events**

**
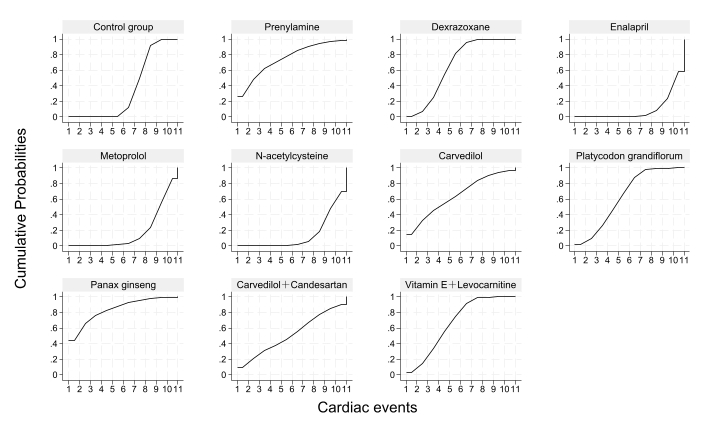
**

**The ratio of mitral annular diastolic peak velocity to atrial systolic velocity (E/A)**

**
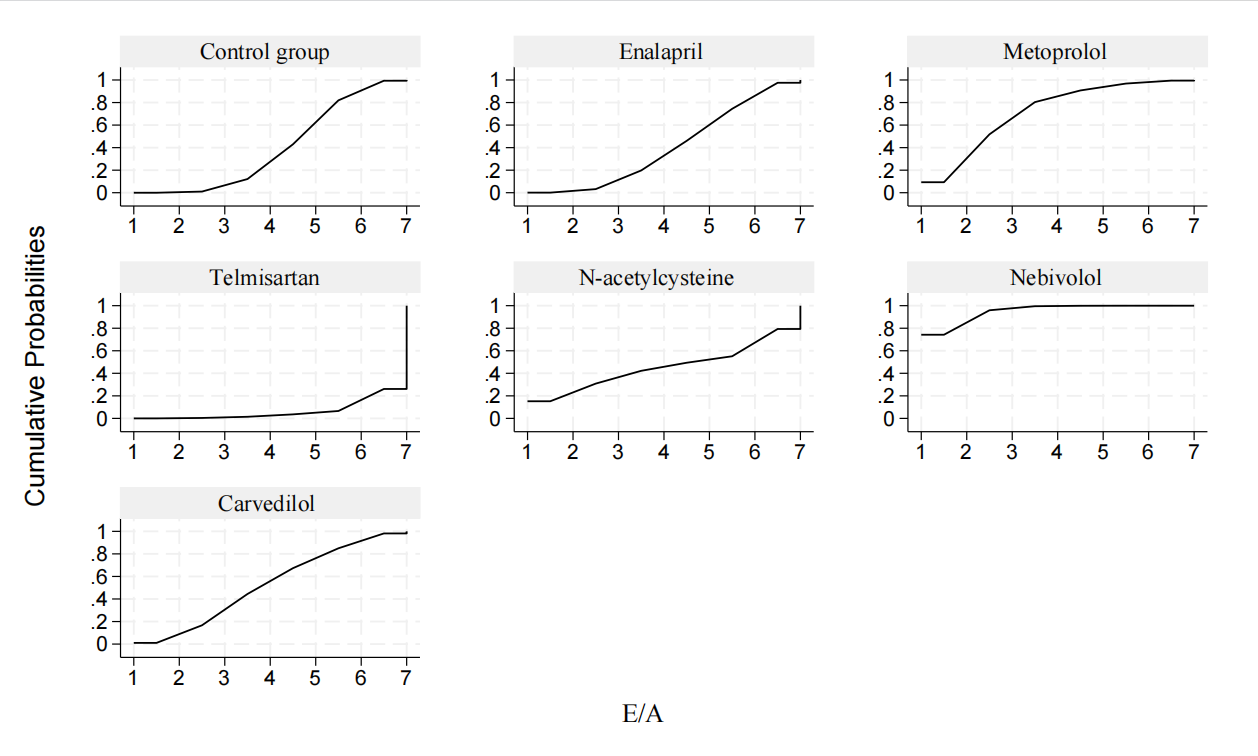
**

**Left ventricular end-diastolic diameter (LVEDD)**

**
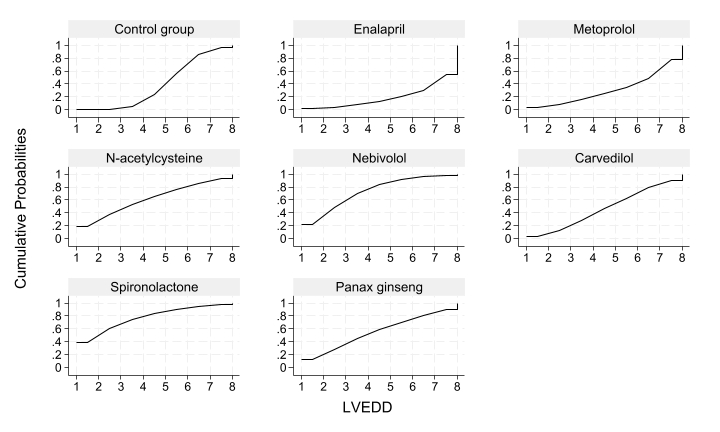
**

**Left ventricular end-systolic diameter (LVESD)**

**
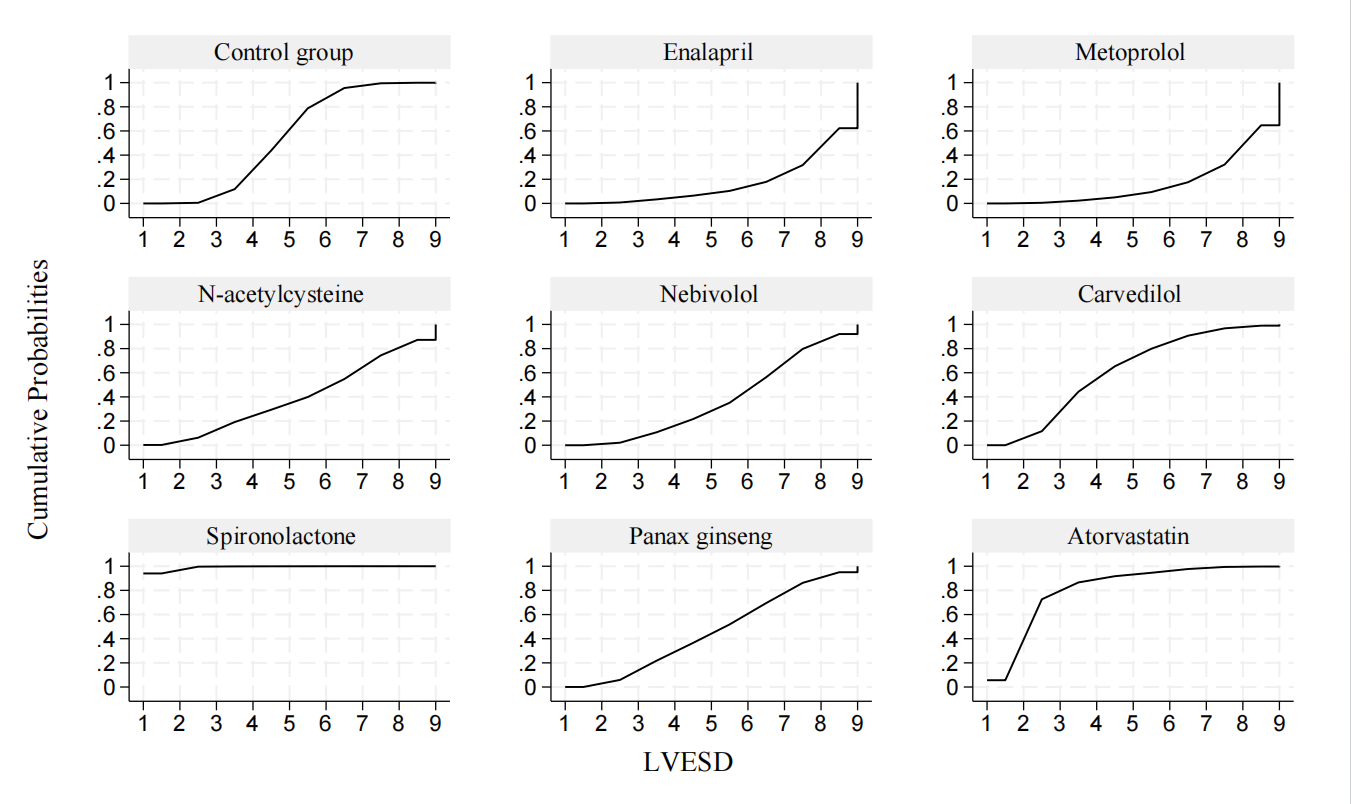
**

**Supplement S9. Comparison-adjusted funnel plots**

**Left ventricular ejection fraction (LVEF)**

**
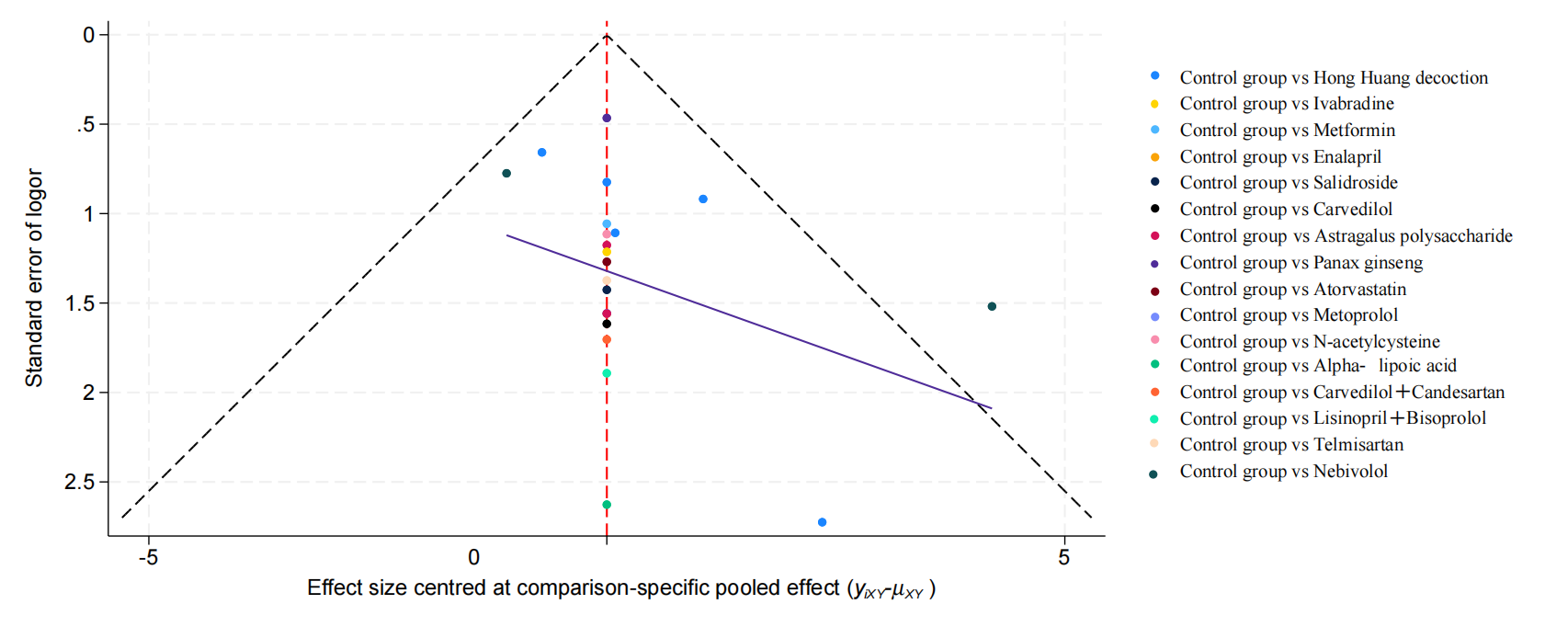
**

**Cardiac events**

**
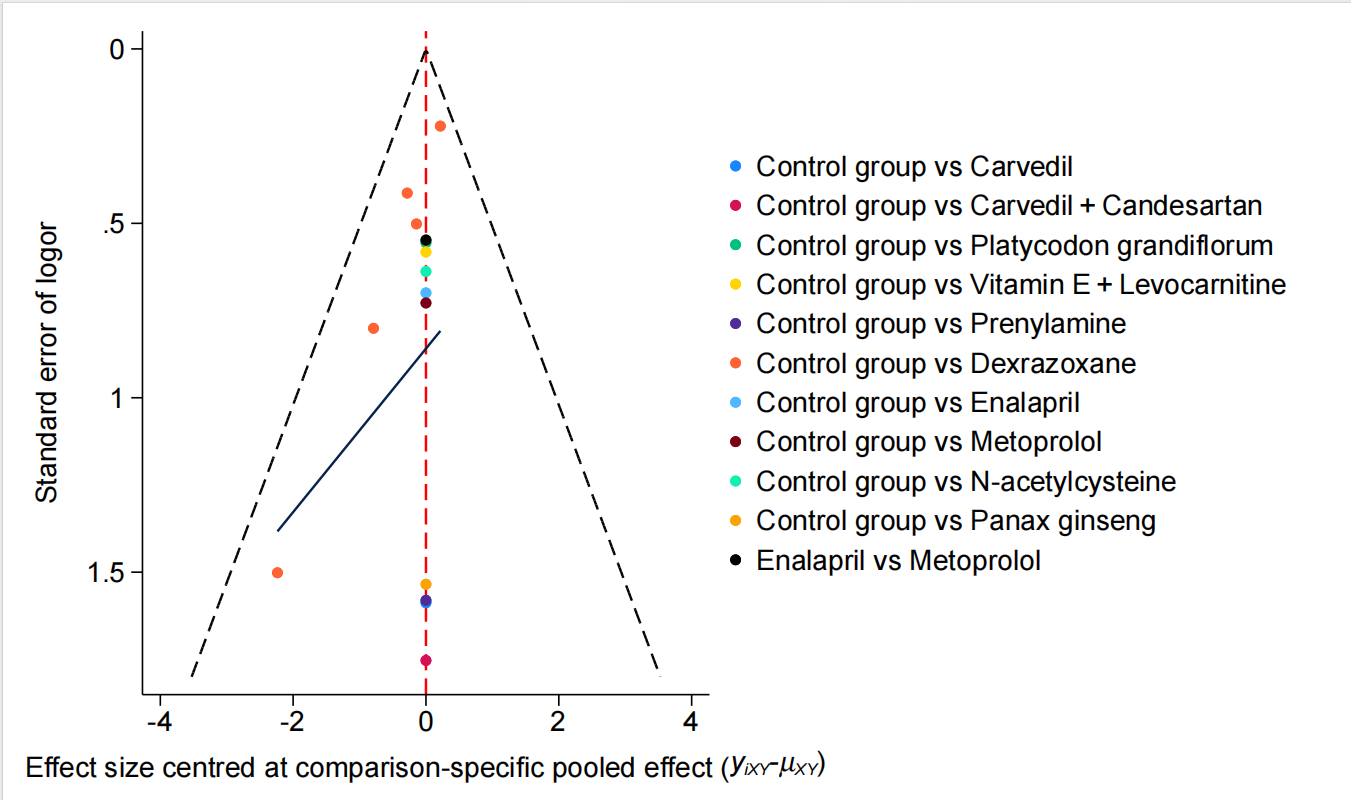
**

**The ratio of mitral annular diastolic peak velocity to atrial systolic velocity (E/A)**

**
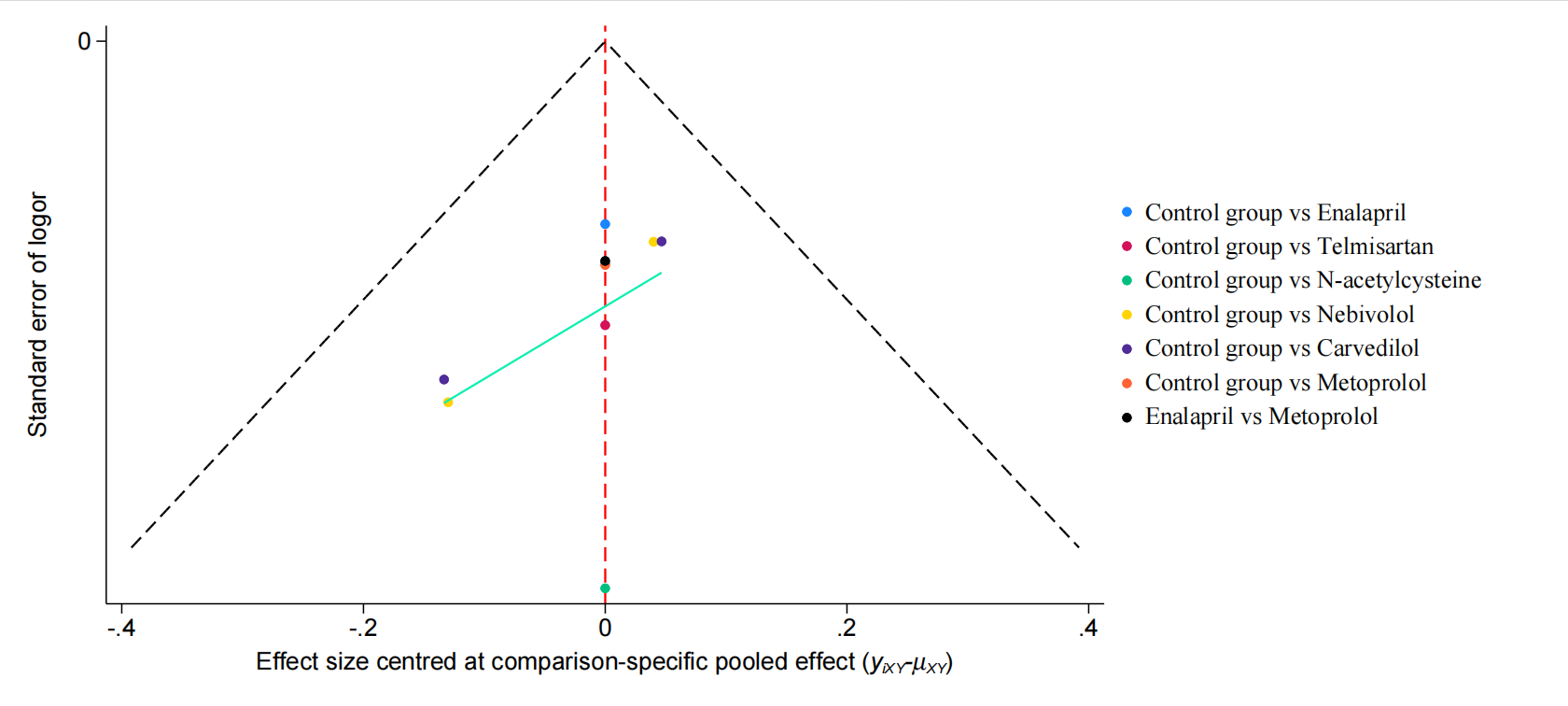
**

**Left ventricular end-diastolic diameter (LVEDD)**

**
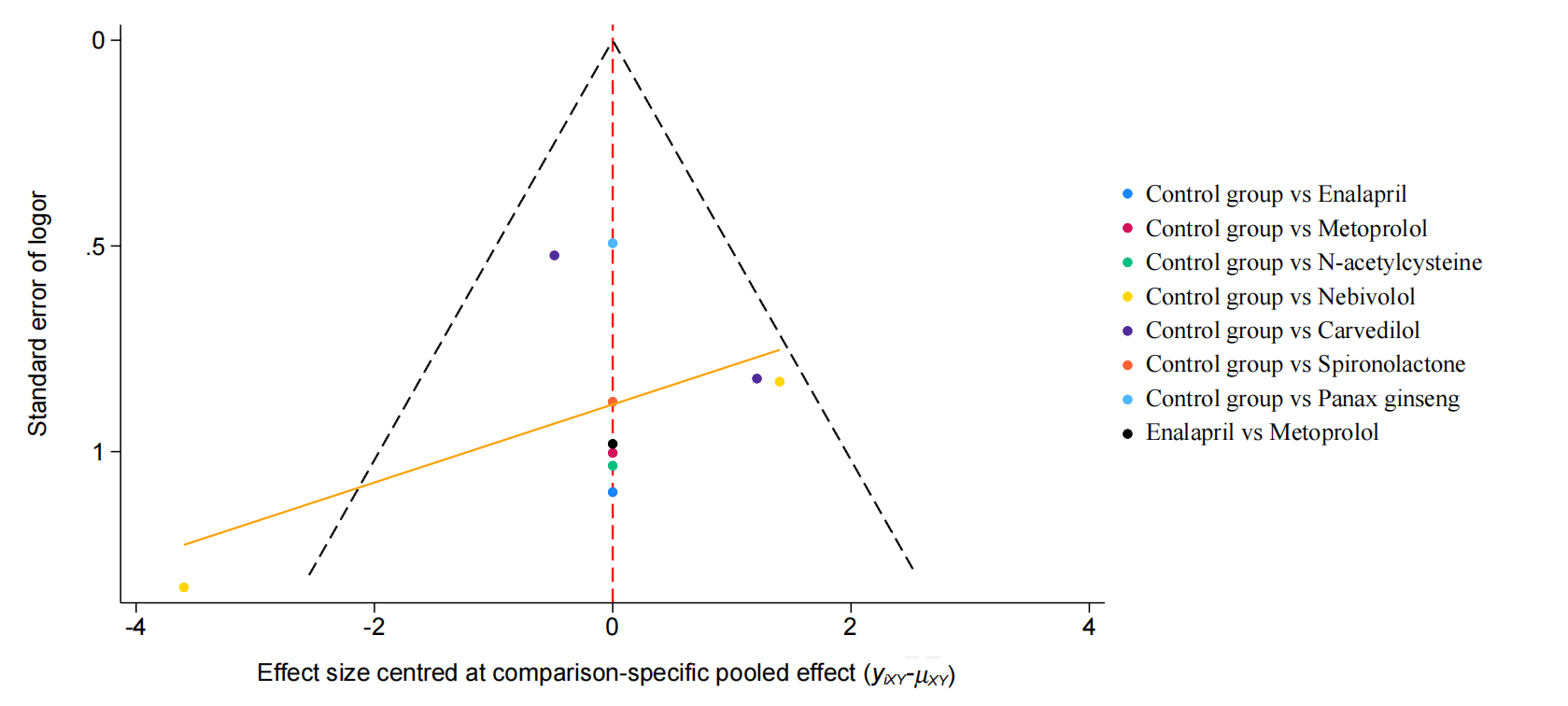
**

**Left ventricular end-systolic diameter (LVESD)**

**
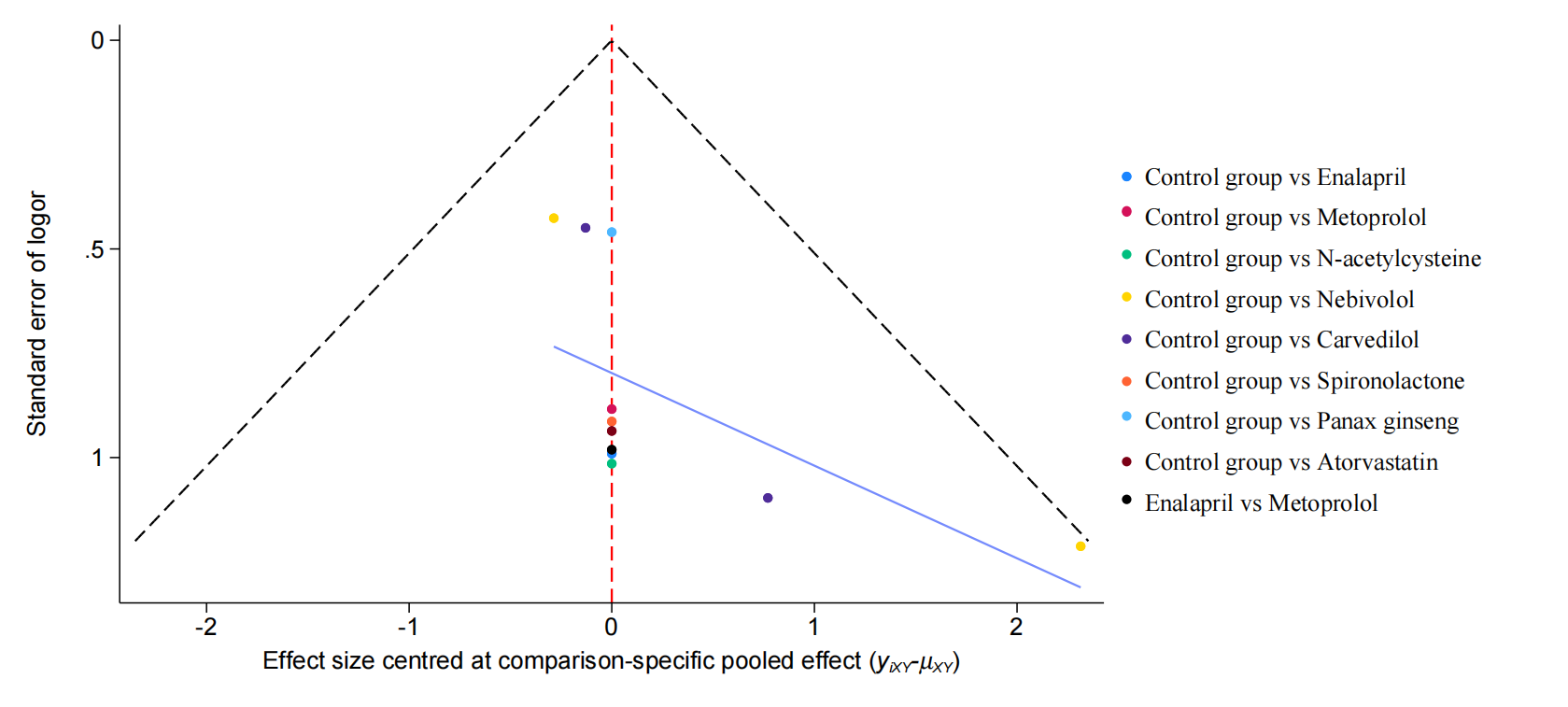
**

**Supplement S10. Pairwise meta-analysis results**

**The ratio of mitral annular diastolic peak velocity to atrial systolic velocity (E/A)**

**
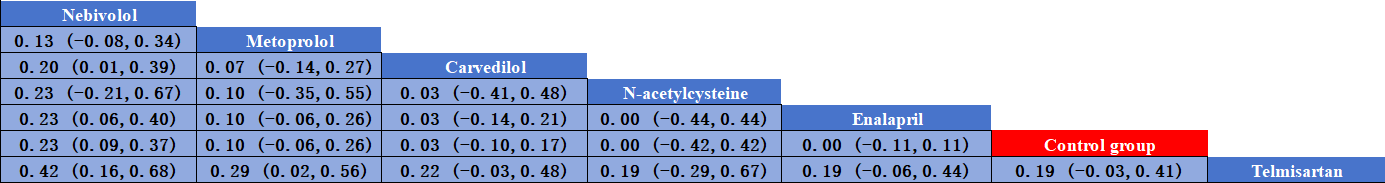
**

**Left ventricular end-diastolic diameter (LVEDD)**

**
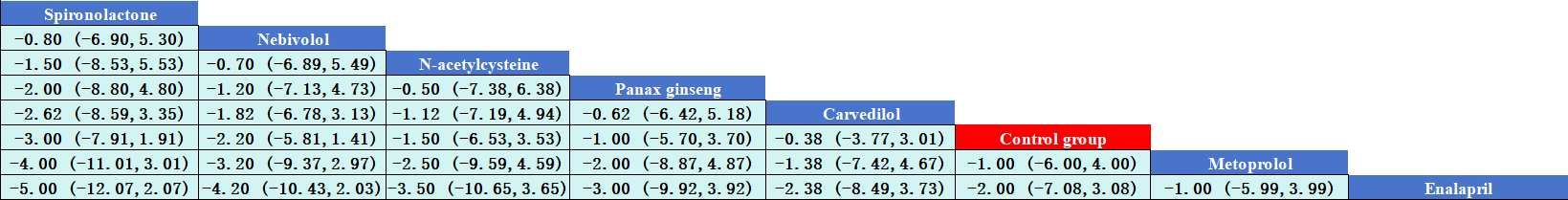
**

**Left ventricular end-systolic diameter (LVESD)**

**
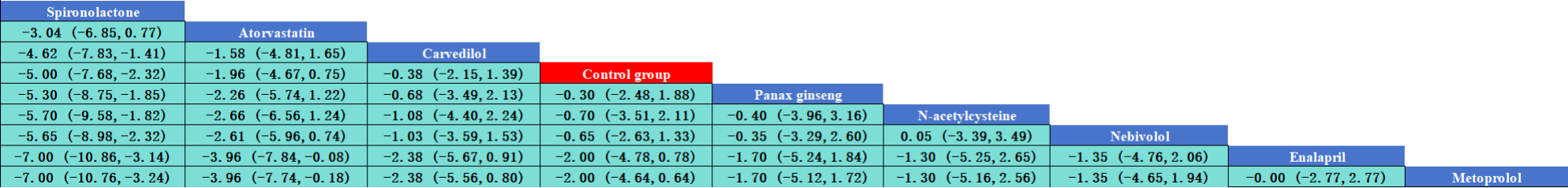
**
